# Do the early social environment and persistent peripartum depressive symptoms shape toddlers' expressive language?

**DOI:** 10.1002/jcv2.12299

**Published:** 2025-02-11

**Authors:** Hsing‐Fen Tu, Linda Forssman, Emma Fransson, Alkistis Skalkidou

**Affiliations:** ^1^ Department of Psychology Uppsala University Uppsala Sweden; ^2^ Department of Women's and Children's Health Uppsala University Uppsala Sweden; ^3^ Department of Microbiology, Tumor and Cell Biology Centre for Translational Microbiome Research Karolinska Institutet Stockholm Sweden

**Keywords:** chronic depressive symptoms, cross‐generational impact, early social environment, infant language development, peripartum depression

## Abstract

**Background:**

Extensive research suggests that peripartum depression is a risk factor for children's early language development. Yet, previous research on this association shows mixed results, often lacking information on the persistence of depression and the social context. This population‐based cohort study addresses this gap by investigating the longitudinal influence of peripartum depressive symptoms on toddlers' expressive language. Specifically, we systematically examined the influences of timing, severity, and persistence of depressive symptoms during pregnancy and the first 6 months postpartum on child expressive language development, while accounting for important social and environmental factors.

**Methods:**

This study is part of a prospective, population‐based investigation conducted within the follow‐up Uppsala Birth Cohort study in Uppsala, Sweden. The final analysis included 2176 mother‐infant dyads (1122 boys, mean age = 18.3 months, SD = 0.7). Perinatal depressive symptoms were assessed at gestational weeks 17 and 32 and at postpartum six weeks and six months, using the Edinburgh Postnatal Depression Scale (cut‐off >12). At 6 months postpartum, mothers were also invited to fill out the Postnatal Bonding Difficulty Questionnaire. At 18 months postpartum, mothers completed the Language Development Survey, which assessed expressive vocabulary and word combinations. Multivariable linear regression models were applied to examine the associations between peripartum depressive symptoms and child language development. Adjusted models incorporated background and social context variables to account for potential confounding factors.

**Results:**

Depressive symptoms during prenatal and postnatal periods were not significantly associated with language outcomes. Our final model identified negative associations with second‐born status, family history of late talkers, countryside residence, and maternal age at childbirth. Positive correlations were found for sex (girl) and pregnancy length. The final model explained 8.4% of the variance (*F*(22, 1566) = 6.525, *p* < 0.001). Furthermore, we found that persistent depressive symptoms were not significantly related to language outcomes (Kruskal‐Wallis test: *H* = 2.227, df = 2, *p* = 0.21).

**Conclusions:**

Our findings found no negative link between peripartum depressive symptoms and expressive language in toddlers, even after considering timing, severity, and persistence. While no immediate direct negative influence of peripartum depressive symptoms was observed, the long‐term cumulative effects later in life remain unclear.

## Highlights

- Previous literature often suggests that peripartum depression might delay infant language development.
- No evidence was found to support a negative association between the timing, severity, and chronicity of depressive symptoms across pregnancy and 6 months postpartum and a toddler's expressive language skills at 18 months of age.
- Biological factors, such as sex and family history of delayed talkers (up to the second degree), were significantly related to a toddler's expressive language skills.
- Birth order and environmental factors (e.g. urban or countryside) might indirectly influence early expressive language due to different life experiences.

Peripartum depression, a form of clinical depression occurring during pregnancy or postpartum (American Psychiatric Association, 2022), affects approximately one in 4–8 women (Mitchell et al., 2023; Woody et al., 2017). Previous evidence has shown that perinatal depressive symptoms may have a negative impact on infant language development (Rogers et al., 2020; Stein et al., 2014) alongside factors such as maternal bonding (Fransson et al., 2020), interaction with siblings (Muñez et al., 2022), and multi‐language exposure (Liberman et al., 2017). In the current retrospective study, we asked whether peripartum depressive symptoms across pregnancy and postpartum were related to a toddler's expressive language while considering various factors in the child's social environment.

Expressive language in early childhood is crucial for facilitating the development of social skills (Gibson et al., 2021; Girard et al., 2017), emotional regulation (Griffiths et al., 2021), and later academic performance (Bleses et al., 2016; Hohm et al., 2007). Emerging evidence suggests that an infant's language development can be shaped by biological (Verhoef et al., 2021) and social‐environmental factors (Hoff, 2006; Rowe & Weisleder, 2020). Given the high prevalence of peripartum depression, extensive research has also focused on the influence of perinatal depressive symptoms on infant language development over the past three decades. However, numerous studies conducted in various countries showed mixed results regarding the association between exposure to peripartum depression and the elevated risk of language delay in childhood (Clifford et al., 2024; Rogers et al., 2020). While some studies focusing on *prenatal exposure* have demonstrated negative impacts on language development (Breen et al., 2018; Donald et al., 2019; Muñoz‐Rocha et al., 2018; Weikum et al., 2012), most studies did not find significant associations (Bandoli et al., 2016; Casper et al., 2003, 2011; Husain et al., 2012; Lin et al., 2017; MacGinty et al., 2020; Murray et al., 2016; Nulman et al., 2012; Rotheram‐Fuller et al., 2018; Tse et al., 2010). The research focused on *postnatal exposure* also showed mixed results. Several studies reported that exposure to postpartum depressive symptoms negatively affected an infant's language development and speech perception during the first year of life (Friedman, 2005; Galler et al., 2000; Kalita, 2010; Kaplan et al., 2012, 2014; Lam‐Cassettari & Kohlhoff, 2020; Quevedo et al., 2012; Schaadt et al., 2022), while other studies investigated language development covering age from the first year to preschool did not find negative associations (Black et al., 2007; Chen et al., 2013; Dagvadorj et al., 2018; Garman et al., 2019; Kurstjens & Wolke, 2001; Piteo et al., 2012). So far, several longitudinal studies have examined the influence of peripartum depressive symptoms on infant language development. Among these, some reported no significant association between peripartum depressive symptoms and language outcomes (Hay & Kumar, 1995; Ibanez et al., 2015; Koutra et al., 2013; O'Leary et al., 2019; Osborne et al., 2018; Parfitt et al., 2014; Pop et al., 1995; Seay, 2019; Senturk, 2017). In contrast, other studies showed mixed results, with varying positive and negative associations at different time points (Brennan et al., 2000; DiPietro et al., 2006; Keim et al., 2011; Moe et al., 2019; Mughal et al., 2018; Punamäki et al., 2018). However, across these studies, only two previous studies observed the exposure across pregnancy to postpartum and demonstrated that the negative impact of peripartum depressive symptoms on an infant's language development may be more related to the persistence rather than the timing of the symptoms (Brennan et al., 2000), or to the number of depressive episodes experienced (Mughal et al., 2019).

While the longitudinal and persistent aspects of depressive symptoms related to infant language development are less explored, there is also a lack of discussion on potential mediators or moderators in this cross‐generational pathway. In the current study, we attempted to explore one potential mediator (maternal bonding) and two key factors, birth order and multilingual exposure at home, as previous evidence suggested that they might influence a child's language development. Maternal bonding refers to a mother's subjective emotional connection, characterized by feelings of warmth, closeness, and attachment to her child (Le Bas et al., 2020). Research from a large cohort study has demonstrated a consistent relationship between perinatal depressive symptoms and bonding failure, spanning from early pregnancy to the first week postpartum (Ohara et al., 2017). Moreover, it has been repeatedly reported that antenatal bonding significantly influences postnatal bonding (Dubber et al., 2015; Rossen et al., 2016; Tichelman et al., 2019). Furthermore, postnatal depressive symptoms have been consistently linked to poor maternal bonding from the early postpartum period to the first year after childbirth (Moehler et al., 2006; O’Higgins et al., 2013). In summary, peripartum depressive symptoms may decrease maternal bonding, which in turn can affect the quality of mother‐child interaction and potentially impact the child's language development. Therefore, investigating the mediating role of maternal bonding is essential for understanding the association between peripartum depressive symptoms and child language outcomes.

Birth order in the current study is used as a proxy to estimate the richness of the social environment, particularly the presence or absence of siblings. It has been proposed that a rich and interactive social engagement with caregivers and siblings can provide an infant with sound context mappings and useful information, which, in turn, influences language development (Kuhl, 2007). During development, infants are able to observe communications from others and form an internal mental process that leads to the production of language (Hoff, 2003). Through social interaction, young children are able to capture and integrate the attentional cues (e.g., temporal contiguity and novelty), social cues (e.g., eye gaze and gestures), and linguistic cues (e.g., rhythm, stress, and intonation) in word learning (Hollich et al., 2000). In the first 2 years of life, siblings, in addition to caregivers, can also provide social interaction for the infant. However, evidence on whether siblings can buffer the potential negative impact of depressive symptoms on infant language development is scarce. Early research demonstrated that compared to first‐born children, later‐born children showed greater conversational skills (Hoff‐Ginsberg, 1998). Conversely, other studies reported that having older siblings might have a negative impact due to competing parent's attention in the social environment (Havron et al., 2019; Schjølberg et al., 2011).

Another key factor related to the social environment we chose was multilingual households. It has been shown that at the age of three, children from multilingual households showed less proficiency in both first and second language compared to children in a monolingual household language advantages (Stow & Dodd, 2003). Previous evidence has demonstrated that, although there was no significant disadvantage for bilingual children, the separation of two languages (e.g., German and English) may be possible at the age of two (Junker & Stockman, 2002), which might contribute to a larger receptive‐expressive gap (the gap between comprehension and production) in early years. A recent review that included 10 empirical studies reported that toddlers exposed to a bilingual environment had a larger expressive vocabulary size compared to children in a monolingual environment (Byers‐Heinlein et al., 2024). Overall, the empirical evidence regarding the influence of multilingual households on language development is inconclusive. Furthermore, to our knowledge, no study has examined the potential interaction between multilingual households and the influence of peripartum depressive symptoms on a toddler's language development.

Finally, although evidence has not yet been replicated, it has been shown that girls have a larger vocabulary (Fenson et al., 1994) and development of word production is on average 3 months earlier than boys (Özçalışkan & Goldin‐Meadow, 2010). Some studies suggest that sex differences may interact with perinatal distress at different times and potentially shape the developmental trajectory (Etchell et al., 2018; Sutherland & Brunwasser, 2018). Therefore, if a significant sex difference in language outcome is observed, we can further explore whether the influence of perinatal depressive symptoms on language is sex‐dependent.

In this study, we investigated whether depressive symptoms and their persistence related to infant expressive language at 18 months, considering various factors. Our four main hypotheses are: (a) toddler language scores will be negatively related to perinatal depressive symptoms, measured by raw scores. This hypothesis examines how levels of depressive symptoms affect toddler language development; (b) the negative association between peripartum depressive symptoms and toddler language will be mediated by maternal bonding. We propose that the effect of depressive symptoms on expressive language development is partially explained by difficulties in maternal bonding; (c) birth order and the presence of multilingual households will be considered as important factors that may influence toddler language development. These variables will be included in our analysis to account for their potential impact on language development; and (d) toddlers of mothers with persistent depressive symptoms, as defined by clinical cut‐off points, will have lower language scores compared to those whose mothers are healthy or experience symptoms only episodically. Overall, we hope our study can provide insights into developmental trajectories and potentially support prevention and intervention efforts.

## METHODS

### Participants and data source

This study is part of the population‐based and prospective cohort study (the Biology, Affect, Stress, Imaging and Cognition (BASIC); Axfors et al., 2019) conducted at the Department of Obstetrics and Gynecology at Uppsala University Hospital in Sweden. The cohort (5492 women, 6478 pregnancies), initiated in 2009, aimed to enhance the understanding of peripartum depression and was followed by the ongoing U‐BIRTH (Uppsala Birth Cohort) project, a follow‐up study, since 2012 (Tu et al., 2023, project website: https://www.ubirth.se/). The BASIC study invited Swedish‐speaking women (>18 years old) scheduled for routine ultrasounds at Uppsala University Hospital. Exclusion criteria included pathological pregnancy and protected personal data. The participation rate was approximately 22% of all women giving birth in Uppsala County. Participants provided signed consent prior to all parts of the study. By the end of 2022, 3525 mother‐child dyads were enrolled in the follow‐up study, and data collection at 18 months postpartum was completed. After filtering out incomplete data, which included missing values for depressive symptom assessment at all four time points and incomplete responses for the language assessment, as well as excluding cases where the child was older than 20 months at the time of submitting the language assessment, 2176 toddlers (1122 boys, 51.6%) were included in the final analysis. This study received approval from the Swedish Ethical Review Authority (the Regional Ethical Review Board in Uppsala) under the reference numbers 2009/171, 2019/01170, and 2022‐03146‐02. All study procedures in the study were conducted in accordance with the 1964 Declaration of Helsinki and fulfilled General Data Protection Regulation requirements for data processing, storage, and security.

### Assessments

#### Assessment of expressive language

When the infant reached 18 months of age, participating mothers were invited to complete the Language Development Survey (LDS; Rescorla & Alley, 2001). The LDS is a parent a report containing 310 words with 14 semantic categories (e.g., animals, vehicles, action words, etc.) to assess a child's expressive vocabulary and the beginning of word combinations. The raw score was determined by evaluating the quantity of words spontaneously used by the child. Recent systematic reviews have shown the LDS to have high predictive validity (Sansavini et al., 2021; Sim et al., 2019). Due to the lack of Swedish norms for the LDS language assessment, we used the raw score (word count) as the dependent variable rather than converting it to a standardized percentile.

#### Assessment of depressive symptoms and persistent levels

Peripartum depressive symptoms were measured using the Swedish version of the Edinburgh Postnatal Depression Scale (EPDS, Cox et al., 1987; Wickberg & Hwang, 1996) during gestational weeks 17 and 32 and at postpartum six weeks and six months. The EPDS consists of 10 questions with a total score ranging from 0 to 30. In the current study, we used a raw score as well as a cut‐off ≥12 to indicate clinically relevant depressive symptoms. This cut‐off point was previously validated in a large Swedish community sample (Wickberg & Hwang, 1996). This four‐time‐point measurement enabled us to employ a within‐person design, allowing us to better isolate and examine the effects of perinatal depression while accounting for variables that remain constant over time. This approach also allowed us to capture both persistent and episodic symptoms. By examining raw scores across the four time points, we assessed the association between depressive symptom levels, timing, and infant language outcome. To explore the influence of persistent depressive symptoms on infant language, we analyzed the continuity of depressive symptoms from pregnancy through 6 months postpartum. Previous research studying persistent postnatal depression defined persistence as symptoms observed across 2 and 8 months postpartum (Netsi et al., 2018). We defined the group with persistent depressive symptoms as women who had three successive time points with an EPDS score higher than the cut‐off point. Overall, we categorized EPDS results into 3 groups representing different persistent levels: healthy, persistent depressive symptoms, and episodic depressive symptoms. In the healthy group, the participant's EPDS score did not reach the cut‐off point at any time point.

#### Mediator and social environment proxies

Maternal bonding was measured at 6 months postpartum using the Postpartum Bonding Questionnaire (PBQ, Brockington et al., 2001). The PBQ consists of 25 questions to assess disorders of the mother‐infant relationship. The total score ranges from 0 to 125, with higher scores indicating more bonding difficulties perceived by the mother. Adequate test‐retest reliability and validity of the PBQ have been reported (Brockington et al., 2006). The PBQ has been previously used in Scandinavian countries and the acceptable internal consistency has been reported (Høivik et al., 2013). Two key factors used as proxies related to social environment at home were considered: birth order (firstborn and only child, firstborn with other siblings, second born, third and fourth born) and the presence of a multilingual household. Please note that information on birth order was documented at birth. For the firstborns, while the majority were the “single child” in our data, some had older siblings, most likely from a previous relationship of the second legal guardian. Therefore, they were coded as “firstborn with other siblings.” However, we did not collect data on the number and ages of these siblings.

#### Covariates

Eleven covariates were selected based on literature, including the infant's sex (Etchell et al., 2018; Schjølberg et al., 2011), the mother's history of smoking (Huizink & Mulder, 2006), the presence of family members (within second degree) with delayed talking (Austerberry et al., 2022), premature birth (Schjølberg et al., 2011), multiple birth (Thorpe, 2006), admission to neonatal intensive care, and pregnancy length (Snijders et al., 2020). Furthermore, we also controlled the mother's age at partus and sociodemographic covariates including the mother's education level, employment, and the child's living area (Rowe & Weisleder, 2020).

### Statistical analysis

#### Data preprocessing

Before testing our hypotheses, we first employed single imputation to handle missing EPDS scores at each time point. If one out of 10 answers were missing, we used mean imputation. If there were multiple missing answers, we coded the observation as not available (NA). Subsequently, to account for missing data across four time points, we performed multiple imputations with five iterations. Besides imputed EPDS scores at each time point, to further explore the relationship between depressive symptoms during pregnancy or postpartum and language, we computed the mean EPDS scores for two distinct periods: during pregnancy and postpartum. We also computed the overall mean EPDS score encompassing both pregnancy and postpartum. By analyzing these different time frames, we attempted to capture the temporal influences of depressive symptoms on toddler language. Next, for classifying different levels of persistent depressive symptoms, the imputed data was further categorized as a binary category at each time point based on the clinical cut‐off point. Finally, given the lack of a normative dataset of the Language Development Survey (LDS) for the Swedish population, we analyzed using the raw word counts. We employed a Quantile‐Quantile (Q‐Q) plot to assess the distribution. If the Q‐Q plot exhibited curvature, indicating deviation from normality, we performed a log transformation to normalize the data.

#### Multivariable linear regression models

We used multivariable linear regression models to examine the association between depressive symptoms (imputed EPDS scores) and child language outcomes. Model 1 included only the key independent variables (imputed EPDS scores from four time points) and the dependent variable (language). Model 2 expanded on Model 1 by incorporating one potential mediator (maternal bonding) and two social environment proxies (birth order and multilingual household). Model 3 further included 11 covariates. We repeated the analysis for all models three times, each with a different independent variable: first using the mean EPDS score during pregnancy, then using the mean EPDS score from the postpartum period, and finally using the overall mean EPDS score across both pregnancy and postpartum periods. Given that previous literature suggests a high correlation between antenatal and postpartum depression (Pearson et al., 2013), we used the variance inflation factor (VIF) to detect multicollinearity. Additionally, we would perform a sex‐stratification analysis built on the final model only if a significant sex difference was observed.

#### Mediator analysis

To analyze the mediation effect, we hypothesized that depressive symptoms influence maternal bonding, which in turn affects language outcomes. We assessed the appropriateness of mediation analysis based on the results from Pearson's correlation between key variables. If the correlations indicated a significant relationship between depressive symptoms, maternal bonding, and language outcomes, we proceeded with mediation analysis using the structural equation modeling method to assess the indirect effects. The mediation model tested whether maternal bonding served as an intermediary factor that partially or fully explained the relationship between depressive symptoms and language development.

#### Kruskal‐Wallis test for examining group differences based on persistent levels

To evaluate whether infants of mothers with persistent depressive symptoms showed significantly lower language scores than infants of healthy mothers or mothers with episodic depressive symptoms, we compared the group differences. Given the expected imbalance between groups according to the prevalence rate of postpartum depression (11.9%) reported in the literature (Woody et al., 2017), which contributes to non‐parametric data, we conducted a Kruskal‐Wallis test. When the test yielded significant results, we used a post hoc analysis (Dunn's test) to identify which groups differed significantly from others.

## RESULTS

### Participant characteristics and descriptive statistics

In Table 1, we present the characteristics of 2176 toddlers (1122 boys, mean age = 18.3 months, SD = 0.69); 1054 girls, mean age = 18.3 months, SD = 0.74) and their mothers. A majority of the mothers held a university degree (78.3%), were employed full‐time at the beginning of their pregnancy (60.5%), and cohabited with the child's second legal guardian (92.7%). Throughout pregnancy and up to 6 months postpartum, 61.2% of the women did not have an Edinburgh Postnatal Depression Scale (EPDS) score above the cut‐off point at any time of measurement. The average EPDS score across four time points ranged from 5.2 to 5.9. Among the 2167 toddlers in our study, the majority were firstborns and the only child at home (*n* = 1346, approximately 61.9% of the sample). Additionally, 400 firstborns had other siblings in the same household. Since the birth order was recorded at birth in the cohort dataset, these additional siblings were most likely older and from the second legal guardian's previous relationship. Only a minority were firstborns from twin births. We did not collect information on those siblings. Moreover, there were 400 second‐born toddlers and 39 third‐ or fourth‐born toddlers. In terms of living environment, 46.9% of the families resided in a big city, 25.6% in a small urban area, and 22.7% in the countryside. Multilingual households accounted for 15.9% of the sample, and 12.1% of the children had family members with a history of delayed talking. For the language assessment, the overall raw mean score was 40.4 (SD = 45.1). Boys had a raw mean score of 33.18 (SD = 38.50, range: 0–265), while girls had a raw mean score of 48.17 (SD = 50.11, range: 0–309). On average, girls had significantly higher raw word counts than boys (t (2174) = −7.850, *p* < 0.001, Cohen's *d* = −0.337). To normalize the data, we applied a log (1 + word count) transformation after visually inspecting the distribution and the Q‐Q plot of the raw word count (see Figure S1). The transformed log values were used as the dependent outcome in the subsequent analyses.

**TABLE 1:** Characteristics of participants included in the study.

Characteristics	Number of available samples	Missing number	Value (mean and SD or % of total available data)
Maternal age at birth (years)	2176	0	31.8 (±4.3)
Child age (months)	2176	0	18.3 (±0.7)
Ethnicity	2120	56	
Swedish	1996		91.7%
Other	124		5.7%
BMI before pregnancy (kg/m^2^)	2056	120	23.7 (±4.2)
Smoking prior to pregnancy	2057	119	
Yes	574		26.4%
Education	2034	142	
University or higher	1704		78.3%
Employment during early pregnancy	2036	140	
Working full‐time	1317		60.5%
Working part‐time/on parental leave/study	628		28.9%
On sick leave/unemployed	91		4.2%
Cohabiting with the second legal guardian	2076	109	
Yes	2018		92.7%
Pregnancy length (days)	2176	0	278.5 (±11.4)
Depressive imputed score (range: 0–30)^a^			
During pregnancy 17 weeks	2028	148	5.2 (±4.4)
During pregnancy 32 weeks	2051	125	5.3 (±4.4)
Postpartum 6 weeks	2041	135	5.9 (±4.6)
Postpartum 6 months	2002	174	5.3 (±4.6)
Persistence of depressive symptoms (cut‐off ≥12)	2484	0	
Healthy	1331		61.2%
Persistent depression	50		2.3%
Episodic depression	795		36.5%
Bonding difficulties (6 months postpartum)	1913	263	8.23 (±6.7, range 0–58)
Infant sex	2176	0	
Boy	1122		51.6%
Birth outcomes			
Infant birth weight (g)	2173	3	3566.8 (±544.8)
Infant birth length (cm)	2166	10	50.7 (±2.4)
Head circumference (cm)	1743	433	34.9 (±1.×6)
Apgar score 5 min	2469	15	9.7 (±0.8)
Admission to intensive care at birth	2075	101	
Yes	210		9.7%
Birth order	2176	0	
First and the only child	1346		61.9%
First but there were more than one at home	400		18.4%
Second‐born	400		18.4%
Third or fourth born	30		1.4%
Twin birth	2176		
Yes	45	0	
Infant living location	2072	104	2.1%
In a big city	1021		46.9%
In a small urban area	558		25.6%
In countryside	493		22.7%
More than one language at home	2027	149	
Yes	345		15.9%
Family member with delayed talking (within second degree relatives)	2028	148	
Yes	263		12.1%
Expressive language word count (boy)	1122	0	33.2 (±38.5, range 0–265)
Expressive language word count (girl)	1054	0	48.2 (±48.1, range 0–309)

Abbreviation: SD, standard deviation.

^a^ Depressive symptoms were measured using the Edinburgh Postnatal Depression Scale with a total score ranging from 0 to 30.

### Associations between peripartum depressive symptoms and language development

Based on the inspection of the raw word counts, the logarithm‐transformed language score was used as an outcome value. In our analysis using multivariable linear regression, we observed that none of the depressive symptoms across all four time points showed significance in Model 1 (Table S1). The collinearity statistics (variance inflation factor, VIF) were all within acceptable limits, suggesting no significant concerns regarding multicollinearity. We found that in Model 2, which includes maternal bonding difficulties, birth order, and the presence of a multilingual household, being second‐born has a significant negative association with the outcome (*ß* = −0.167, *p* = 0.02, see Table S1). Table 2 summarizes the final model (Model 3). In this model, the “second‐born” variable remains significantly negative (*ß* = −0.172, *p* = 0.022). Furthermore, several other factors significantly contribute to negative associations with the outcome. These include having a family history of late talkers (*ß* = −0.187, *p* = 0.026), living in small urban areas (*ß* = −0.225, *p* < 0.001) or rural areas (*ß* = −0.288, *p* < 0.001), and the mother's age at childbirth (*ß* = −0.022, *p* < 0.001). Interestingly, girls (*ß* = 0.404, *p* < 0.001) and longer pregnancy length (*ß* = 0.010, *p* < 0.001) are positively correlated with the outcome. All reported *ß* coefficients are unstandardized to directly show the effect of a one‐unit change in the independent variable on the dependent variable. Overall, the final model (Model 3) suggests that approximately 8.4% of the variance in the outcome variable can be explained by the model. For the significant categorical variables in Model 3, we performed additional independent *t*‐tests and analyses of variance (ANOVA) to compare differences in raw word count. These analyses were not part of the original statistical plan, and the results are presented in Table S2 to provide comprehensive information.

**TABLE 2:** Final model summary of multivariable linear regression of perinatal depressive symptoms (four time points) and toddler expressive language development.

Variable	Model 3 (final model)		
	*F*(22, 1566) = 6.525, *p* < 0.001, *R* ^2^ = 0.084		
	*β*	95% CI	VIF
EPDS 17th week of pregnancy	0.015	[−0.002, 0.032]	1.367
EPDS 32nd week of pregnancy	−0.003	[−0.021, 0.015]	1.432
EPDS 6 weeks postpartum	−8.3 × 10^−4^	[−0.017, 0.015]	1.384
EPDS 6 months postpartum	−0.002	[−0.011, 0.007]	1.134
Maternal bonding difficulties (6 months postpartum)	−0.002	[−0.013, 0.006]	1.132
Birth order (first born with other siblings)	0.031	[−0.114, 0.177]	1.024
Birth order (second born)	−0.172*	**[−0.318, −0.025]**	
Birth order (third or fourth born)	−0.194	[−0.695, 0.307]	
Multilingual household (yes)	−0.083	[−0.230, 0.064]	1.023
Sex (girl)	0.404***	**[0.296, 0.512]**	**1.011**
Preterm birth (yes)	0.642	[−0.152, 1.436]	1.114
Admission to NICU at birth (yes)	−0.094	[−0.282, 0.094]	1.069
Twin birth (yes)	−0.284	[−0.635, 0.066]	1.038
Family member with delayed talking (yes)	−0.187*	**[−0.351, −0.022]**	**1.015**
Living area (small urban area)	−0.225***	**[−0.356, −0.093]**	**1.019**
Living area (countryside)	−0.288***	**[−0.425, −0.151]**	
Mother's age at partus	−0.022***	**[−0.035, −0.009]**	**1.050**
Pregnancy length	0.010***	**[0.004, 0.015]**	**1.169**
Mother's education (university)	0.124	[−0.036, 0.284]	1.083
Mother's employment (full‐time)	0.003	[−0.273, 0.279]	1.032
Mother's employment (part‐time or on leave)	0.015	[−0.269, 0.299]	
Mother's history of smoking (yes)	0.112	[−0.013, 0.236]	1.041

*Note*: Regression formular: Model 3_(H0)_ = 3.227 + 0.015 (EPDS 17th week of pregnancy) − 0.003 (EPDS 32nd week of pregnancy) − 8.264 × 10^−4^ (EPDS 6 weeks postpartum) − 0.002 (EPDS 6 months postpartum) − 0.002 (Maternal bonding difficulties) + 0.031 (Birth order, First) − 0.172 (Birth order, Second) − 0.194 (Birth order, Third and fourth) − 0.083 (Multilingual household, Yes) + 0.404 (Sex, Girl) + 0.642 (Preterm birth, Yes) − 0.094 (Admission to NICU at birth, Yes) − 0.284 (Twin birth, Yes) − 0.187 (Family member with delayed talking, Yes) − 0.225 (Living area, in a small urban area) − 0.288 (Living area, In the countryside) − 0.022 (Mother's age at partus) + 0.010 (Pregnancy length) + 0.124 (Mother's education, University) + 0.003 (Mother's employment, Working full‐time) + 0.015 (Mother's employment, Working part−time/On parental leave/Studying) + 0.112 (Mother's history of smoking, Yes).

Abbreviations: CI, confidence interval; EPDS, The Edinburgh postnatal depression scale; NICU, neonatal intensive care unit; VIF, variance inflation factor.

*p < 0.05, **p < 0.01, ***p < 0.001. *β* is unstandardized. Bold text denotes statistically significant results.

Birth order: reference, firstborn and single child is the reference. Living area: “in a big city” is the reference.

We analyzed average EPDS scores across different periods in three models. For prenatal periods (17 and 32 weeks), refer to Table S3; for postnatal periods (6 weeks and 6 months), refer to Table S4; and for the combined EPDS mean across all four time points, refer to Table S5. Overall, across all models, EPDS scores averaged over various timeframes did not alter the findings significantly. However, the initially observed negative association with being second‐born did not retain statistical significance after accounting for all covariates, particularly when using either the postpartum mean EPDS score or the mean EPDS score across all four time points.

### Language outcome across groups with different levels of persistent depressive symptoms

Overall, there were 50 women (2.3% of the sample) with three EPDS scores higher than the cuff‐off successively, while the majority of women (*n* = 1331, 61.2%) never reported EPDS scores higher than the cut‐off across pregnancy and 6 months postpartum. Using a Kruskal‐Wallis test, a non‐parametric test, the result indicates that the levels of persistent depressive symptoms did not have a statistically significant effect on the outcome (*H* = 2.277, df = 2, *p* = 0.320).

### Relationships between key variables, hypothesized mediator, and two proxies for social environment

The levels of maternal bonding difficulties were hypothesized to mediate the negative impact of peripartum depression on infant language. However, since it was significantly related only to depressive symptoms and not to language outcomes, the assumption for testing a mediator was not fulfilled (see Figure S2). Therefore, we did not proceed with the mediator analysis. For two proxies for the social environment at home, the presence of a multilingual household was not related to the language outcome. However, birth order, specifically being the second‐born, was significantly related to the outcome across all models. Notably, the coefficient for firstborns with siblings showed a positive trend, although it does not reach statistical significance. Like‐wise, coefficients for third and fourth‐born children are negative, also without statistical significance.

### Results of the sex‐stratification analysis

As reported earlier, the average word count for girls was significantly higher than that for boys. To further investigate these differences, we conducted a sex‐stratification analysis based on Model 3 reported in Table 2 (see Tables S6 and S7 for additional details). This analysis revealed that the disadvantage associated with being a second‐born child became insignificant for girls and approached significance for boys (*p* = 0.051). We also found that the negative association between having a family history of delayed speech and the outcome remained significant only for girls, not boys. Additionally, like the Model 3 reported in Table 2, several covariates, including having a family member with late speech, living in a small urban area, residing in the countryside, and the mother's age at childbirth, continued to show a significant negative influence for both girls and boys. To assess the adequacy of our sample sizes for detecting significant effects in our multivariable linear regression models, we additionally conducted post‐hoc power analyses. In the subgroup analyses, the power was approximately 0.981 for girls (effect size *f*2 = 0.075) and around 0.933 for boys (effect size *f*2 = 0.060).

## DISCUSSION

This study investigated the associations between depressive symptoms across pregnancy and 6 months postpartum, and toddler expressive language at 18 months. In line with the recent systematic review by Clifford et al. (2024), our findings did not support the detrimental impact of peripartum depressive symptoms on expressive language in toddlerhood. Furthermore, there was no significant difference in expressive language between toddlers of healthy mothers, mothers with episodic depressive symptoms, or mothers with persistent depressive symptoms. We also explored the potential mediator and tested two social environment proxies suggested in the literature. However, our findings did not provide evidence that maternal bonding difficulties and the presence of multilingual households influenced the relationship between peripartum depressive symptoms and language development. Intriguingly, while peripartum depressive symptoms alone are not linked to toddler expressive language outcomes, factors such as birth order, sex, living areas, mother's age at partus, and pregnancy length play significant roles.

The non‐significant results observed in our study could be due to several plausible explanations. One possible reason is that the adverse impact of peripartum depression does not exist, or that its cumulative effects only become evident in later childhood. Second, given most participating parents in our samples hold a university degree (or higher) and Uppsala is a university town, the high level of education and resources available to these families may have mitigated any potential negative effects of peripartum depressive symptoms on toddler language development.

Third, by focusing on depressive symptoms in this cross‐generational pathway, we may have overlooked the influences of other peripartum mental distress, such as anxiety. In other words, though we included multiple time points, we were not able to capture the full picture of the complex dynamics involving other psychological factors. For instance, as a recent meta‐analysis reported by Rogers et al., depressive symptoms alone were not related to infant language development. However, anxiety symptoms alone or with depressive symptoms in the perinatal phase might be related to language delay (Rogers et al., 2020). Given the high comorbidity of depression and anxiety (Ramakrishna et al., 2019), the complexity between timing, severity, and how these factors interact with biological, social, and other environmental factors is still less known.

Another possible explanation is that the negative impact is mitigated by the rich social interaction effect. For example, an infant's ability to follow others' gaze in face‐to‐face interactions plays a crucial role in facilitating language development (Tenenbaum et al., 2015; Çetinçelik et al., 2021). However, depressed mothers might not engage with their infants and look away from them more compared to non‐depressed mothers (Tronick & Reck, 2009). Research also indicates that higher levels of postpartum depressive symptoms were associated with decreased gaze following in infants (Astor et al., 2020). Nevertheless, these findings were not observed in a social group where young infants were cared for by parents as well as extended family members (Astor et al., 2022), suggesting that the rich and high‐quality social interactions between infants and adults may provide protection against the negative effects of peripartum depression. In Sweden, infants typically start attending daycare at 1 year of age, so attendance might have buffered effects of peripartum depression. Our language assessment took place when a child reached the age of 18 months. Although we collected data on social exposure to peer groups in daycare centers, we lack information on the quantity and quality of social interaction infants have with extended family and other social groups.

Regarding one potential mediator (maternal bonding difficulties), our data did not meet the necessary assumptions for conducting mediator analysis. Despite maternal bonding difficulties not showing a significant association with the language outcome, we observed a significant positive correlation with depressive symptoms across all four time points. The results are in line with previous evidence suggesting that peripartum depression increases the risk of early maternal bonding difficulties (Slomian et al., 2019) and poor maternal‐infant interaction (Lam‐Cassettari & Kohlhoff, 2020), which in turn may contribute to lower levels of maternal sensitive parenting (Bernard et al., 2018). While our study provides insights into these relationships, the interaction between maternal bonding difficulties, depression, and later child language development remains unclear. Future research should explore this area further to understand the long‐term impacts on child outcomes. Such insights could inform interventions focused on improving early maternal‐infant interactions and supporting child language development.

Surprisingly, our results indicate potential disadvantages for second‐born children compared to firstborns in expressive language development at 18 months of age. This finding aligns with previous research findings (Havron et al., 2019; Schjølberg et al., 2011), suggesting that birth order may play a role in language development. However, it may not simply be due to the competition between siblings for parents' limited resources to attend to their children according to our data. In our sample, there were 400 firstborns with other (potentially older) siblings, likely from a previous relationship of the second legal guardian, showing the highest average word count compared to only child, second born, or third or fourth born. Unfortunately, we did not collect information on the number or ages of these siblings and the quality of their daily interaction, which might influence the language outcome of our study participants. Therefore, it is difficult to compare whether firstborns with older siblings received more attention compared to second‐born and only children, potentially leading to more opportunities to advance their expressive language. Moreover, following sex‐stratification analysis, the disadvantages associated with being a second‐born only approached significance in boys, suggesting that sex differences may influence the impact of birth order on expressive language development. However, although our power analyses indicate that we had adequate power to detect significant effects, it is important to acknowledge that smaller or more subtle effects might still go undetected. The absence of significant findings for variables such as being a second‐born or having a family history of delayed speech may be attributed to their relatively small effect sizes or other methodological factors. Nevertheless, using birth order or household size as indicators to measure social interactions at home with peers may oversimplify the intricate dynamics of early childhood development. Future research may benefit from employing both quantitative and qualitative methodologies to comprehensively explore these relationships and better understand the nuances of familial and social influences on language acquisition and development.

Furthermore, our findings show that parents reported instances of delayed speech within the family, typically in one of the parents or the child's siblings, the child might exhibit a tendency to commence talking later compared to children without a family member with a history of delayed speech. In fact, it has previously been reported in a large‐scale study showing that there is a moderate heritability not only in expressive language but also in receptive language, grammar, phonology, articulation, lexical knowledge, and verbal memory in childhood (Kovas et al., 2005). However, as a covariate in our analyses, these familial influences need to be carefully considered to accurately assess their impact on language development.

Another interesting finding is that, in comparison to boys, our results showed that the average word count of girls' is higher and the variability is larger than boys. Previous research has indicated a small advantage for girls over boys in language production (Eriksson et al., 2012; Rinaldi et al., 2023). However, language production in infancy can be influenced by various factors, such as culture and birth order. In the context of Swedish‐speaking children, existing evidence provides mixed results regarding language skills (Berglund & Eriksson, 2000; Berglund et al., 2005). It is commonly believed that there is, to some extent, sex difference in language development. Some evidence suggests that sex differences might interact with perinatal distress at different times and potentially shape the developmental trajectory (Etchell et al., 2018; Sutherland & Brunwasser, 2018), which we did not observe in our data. Future research might be interested in combining language assessments in other ecologically validated contexts together with a parent report.

Lastly, we observed that the residential location of the family might be related to early expressive language. Compared to toddlers living in a big city, those in small urban areas or the countryside had lower language outcomes. In big cities, infants likely benefit from increased exposure to various forms of social interaction. Taking the population density as an example, in Sweden, the population density by country ranges from 2.6 inhabitants/km^2^ to 374.6 inhabitants/km^2^, which is 48.9 inhabitants/km^2^ in the county for the current study population (Dyvik, 2023). Daily exposure to social context and intensity might vary from city to countryside. However, it is important to note that in our study, geographical area served as a proxy to approximate broader social environments and socioeconomic statuses. Further research is warranted to explore whether different living environments (city, small urban area, countryside) provide varying social dynamics or resources that influence language development.

## STRENGTHS AND LIMITATIONS

The current study employed a large, detailed, and longitudinal dataset to investigate the relationship between peripartum depressive symptoms and a toddler's expressive language. This large cohort gave us an opportunity to establish a high‐resolution timeframe to examine the levels of chronicity peripartum depressive symptoms and their relationship with child outcome. Specifically, our study's within‐person design includes multiple measures of depressive symptoms at 17 weeks, 32 weeks during pregnancy, and at 6 weeks and 6 months postpartum. This approach allowed us to more precisely control for time‐invariant factors related to perinatal depression, thereby enhancing the internal validity of our findings by minimizing the influence of these constant factors on our results. Unlike monozygotic twin studies, which adjust only for genetic and shared environmental factors, our design also accounted for variations in individual and environmental risks, such as the number of siblings, multilingual households, and a broader environment (e.g., living area). There are several limitations that might restrict the generalizability of our findings. First, our sample primarily consisted of participants with higher education levels and socioeconomic status which are seen to be protective factors of language development (Pace et al., 2017). Second, most of the data included relied on parent reports. Future research might benefit from longitudinal, behavioral observation, and structural or semi‐structural interviews of parents. Third, while we used birth order as a proxy for the number of siblings and the richness of the home social environment, we were surprised by the significant proportion of firstborns (documented at birth) who had siblings. Due to our lack of detailed information on the actual number and ages of these siblings, we are unable to fully assess how sibling dynamics might influence developmental outcomes. Future research should incorporate detailed data on sibling characteristics and the quality of home social interactions to more accurately evaluate their impact on developmental outcomes. Fourth, our study did not include any information on the second legal guardian of the infant. The physical and mental health states, education, and other factors might also affect mothers' mental health and infants' development. Parental depression was reported to have a significant impact on a child's language development (Malin et al., 2012; Paulson et al., 2009). Fifth, it must be noted that our study specifically focused on the impact of depressive symptoms and not on clinically diagnosed depressive episodes, so results may not be generalizable to associations with depressive episodes. Future research might be interested in further studying the interaction between peripartum depressive symptoms and other types of mental distress, as well as a more comprehensive approach investigating a wide range of social and environmental influences, focusing on eventual protective factors. Lastly, the current study is a sub‐study of a larger cohort study and was not preregistered. Given the extensive range of variables and analyses involved, preregistration could have substantially improved both the planning and execution.

## CONCLUSION

In conclusion, our findings did not provide evidence supporting a negative association between peripartum depressive symptoms and expressive language development in toddlers. Additionally, we did not find that varying levels of persistent depressive symptoms played a significant role in this cross‐generational pathway. Nonetheless, our observations highlight the presence of several factors–including sex, birth order, and residential areas–that may potentially influence language development, suggesting the need for further investigation into these complex interactions.

## AUTHOR CONTRIBUTIONS

**Hsing‐Fen Tu:** Formal analysis; Funding acquisition; Investigation; Methodology; Project administration; Visualization; Writing ‐ original draft; Writing ‐ review and editing. **Linda Forssman:** Supervision; Writing ‐ review and editing. **Emma Fransson:** Funding acquisition; Supervision; Writing ‐ review and editing. **Alkistis Skalkidou:** Conceptualization; Data curation; Funding acquisition; Methodology; Supervision; Writing ‐ review and editing.

## CONFLICT OF INTEREST STATEMENT

All authors declare that there is no conflict of interest in this study.

## ETHICAL CONSIDERATIONS

This study received approval from the Swedish Ethical Review Authority (the Regional Ethical Review Board in Uppsala) under the reference numbers 2009/171, 2019/01,170, and 2022‐03146‐02. All procedures in the current study were conducted in accordance with the 1964 Declaration of Helsinki and fulfilled General Data Protection Regulation requirements for data processing, storage, and security. Participants provided signed consent prior to all parts of the study.

## Supporting information

Supporting Information S1

## Data Availability

The analytic codes necessary to reproduce the analyses presented here are available upon request.

## References

[jcv212299-bib-0001] Association, A. P. (2022). Diagnostic and statistical manual of mental disorders: DSM‐5TR (Vol. 5). American Psychiatric Association Publishing.

[jcv212299-bib-0002] Astor, K. , Lindskog, M. , Forssman, L. , Kenward, B. , Fransson, M. , Skalkidou, A. , Tharner, A. , Cassé, J. , & Gredebäck, G. (2020). Social and emotional contexts predict the development of gaze following in early infancy. Royal Society Open Science, 7(9), 201178. 10.1098/rsos.201178 33047063 PMC7540771

[jcv212299-bib-0003] Astor, K. , Lindskog, M. , Juvrud, J. , Namgyel, S. C. , Wangmo, T. , Tshering, K. , Gredebäck, G. , & Gredebäck, G. (2022). Maternal postpartum depression impacts infants’ joint attention differentially across cultures. Developmental Psychology, 58(12), 2230–2238. 10.1037/dev0001413 36107661

[jcv212299-bib-0004] Austerberry, C. , Mateen, M. , Fearon, P. , & Ronald, A. (2022). Heritability of psychological traits and developmental milestones in infancy: A systematic review and meta‐analysis. JAMA Network Open, 5(8), e2227887. 10.1001/jamanetworkopen.2022.27887 35994288 PMC9396365

[jcv212299-bib-0005] Axfors, C. , Bränn, E. , Henriksson, H. E. , Hellgren, C. , Kallak, T. K. , Fransson, E. , Lager, S. , Iliadis, S. I. , Sylvén, S. , Papadopoulos, F. C. , Ekselius, L. , Sundström‐Poromaa, I. , & Skalkidou, A. (2019). Cohort profile: The Biology, affect, stress, imaging and cognition (BASIC) study on perinatal depression in a population‐based Swedish cohort. BMJ Open, 9(10), e031514. 10.1136/bmjopen-2019-031514 PMC6830667 31641004

[jcv212299-bib-0006] Bandoli, G. , Coles, C. D. , Kable, J. A. , Wertelecki, W. , Granovska, I. V. , Pashtepa, A. O. , & Chambers, C. D. , & CIFASD . (2016). Assessing the independent and joint effects of unmedicated prenatal depressive symptoms and alcohol consumption in pregnancy and infant neurodevelopmental outcomes. Alcoholism: Clinical and Experimental Research, 40(6), 1304–1311. 10.1111/acer.13081 27129610 PMC4889502

[jcv212299-bib-0007] Berglund, E. , & Eriksson, M. (2000). Communicative development in Swedish children 16‐28 months old: The Swedish early communicative development inventory—Words and sentences. Scandinavian Journal of Psychology, 41(2), 133–144. 10.1111/1467-9450.00181 10870432

[jcv212299-bib-0008] Berglund, E. , Eriksson, M. , & Westerlund, M. (2005). Communicative skills in relation to gender, birth order, childcare and socioeconomic status in 18‐month‐old children. Scandinavian Journal of Psychology, 46(6), 485–491. 10.1111/j.1467-9450.2005.00480.x 16277649

[jcv212299-bib-0009] Bernard, K. , Nissim, G. , Vaccaro, S. , Harris, J. L. , & Lindhiem, O. (2018). Association between maternal depression and maternal sensitivity from birth to 12 months: A meta‐analysis. Attachment and Human Development, 20(6), 578–599. 10.1080/14616734.2018.1430839 29374991

[jcv212299-bib-0010] Black, M. M. , Baqui, A. H. , Zaman, K. , McNary, S. W. , Le, K. , Arifeen, S. E. , Hamadani, J. D. , Parveen, M. , Yunus, M. , & Black, R. E. (2007). Depressive symptoms among rural Bangladeshi mothers: Implications for infant development. Journal of Child Psychology and Psychiatry, 48(8), 764–772. 10.1111/j.1469-7610.2007.01752.x 17683448

[jcv212299-bib-0011] Bleses, D. , Makransky, G. , Dale, P. S. , Højen, A. , & Ari, B. A. (2016). Early productive vocabulary predicts academic achievement 10 years later. Applied PsychoLinguistics, 37(6), 1461–1476. 10.1017/S0142716416000060

[jcv212299-bib-0012] Breen, M. S. , Wingo, A. P. , Koen, N. , Donald, K. A. , Nicol, M. , Zar, H. J. , Ressler, K. J. , Buxbaum, J. D. , & Stein, D. J. (2018). Gene expression in cord blood links genetic risk for neurodevelopmental disorders with maternal psychological distress and adverse childhood outcomes. Brain, Behavior, and Immunity, 73, 320–330. 10.1016/j.bbi.2018.05.016 29791872 PMC6191930

[jcv212299-bib-0013] Brennan, P. A. , Hammen, C. , Andersen, M. J. , Bor, W. , Najman, J. M. , & Williams, G. M. (2000). Chronicity, severity, and timing of maternal depressive symptoms: Relationships with child outcomes at age 5. Developmental Psychology, 36(6), 759–766. 10.1037/0012-1649.36.6.759 11081699

[jcv212299-bib-0014] Brockington, I. F. , Fraser, C. , & Wilson, D. (2006). The postpartum bonding questionnaire: A validation. Archives of Women's Mental Health, 9(5), 233–242. 10.1007/s00737-006-0132-1 16673041

[jcv212299-bib-0015] Brockington, I. F. , Oates, J. , George, S. , Turner, D. , Vostanis, P. , Sullivan, M. , Loh, C. , & Murdoch, C. (2001). A screening questionnaire for mother‐infant bonding disorders. Archives of Women's Mental Health, 3(4), 133–140. 10.1007/s007370170010

[jcv212299-bib-0016] Byers‐Heinlein, K. , Gonzalez‐Barrero, A. M. , Schott, E. , & Killam, H. (2024). Sometimes larger, sometimes smaller: Measuring vocabulary in monolingual and bilingual infants and toddlers. First Language, 44(1), 74–95. 10.1177/01427237231204167 38283538 PMC10810733

[jcv212299-bib-0017] Casper, R. C. , Fleisher, B. E. , Lee‐Ancajas, J. C. , Gilles, A. , Gaylor, E. , DeBattista, A. , & Hoyme, H. E. (2003). Follow‐up of children of depressed mothers exposed or not exposed to antidepressant drugs during pregnancy. The Journal of Pediatrics, 142(4), 402–408. 10.1067/mpd.2003.139 12712058

[jcv212299-bib-0018] Casper, R. C. , Gilles, A. A. , Fleisher, B. E. , Baran, J. , Enns, G. , & Lazzeroni, L. C. (2011). Length of prenatal exposure to selective serotonin reuptake inhibitor (SSRI) antidepressants: Effects on neonatal adaptation and psychomotor development. Psychopharmacology, 217(2), 211–219. 10.1007/s00213-011-2270-z 21499702

[jcv212299-bib-0019] Çetinçelik, M. , Rowland, C. F. , & Snijders, T. M. (2021). Do the eyes have it? A systematic review on the role of eye gaze in infant language development. Frontiers in Psychology, 11, 589096. 10.3389/fpsyg.2020.589096 33584424 PMC7874056

[jcv212299-bib-0020] Chen, H. H. , Hwang, F. M. , Wang, K. L. , Chen, C. J. , Lai, J. C. Y. , & Chien, L. Y. (2013). A structural model of the influence of immigrant mothers' depressive symptoms and home environment on their children's early developmental outcomes in Taiwan. Research in Nursing and Health, 36(6), 603–611. 10.1002/nur.21566 24242197

[jcv212299-bib-0021] Clifford, B. N. , Rainey, V. , & Eggum, N. D. (2024). Parental postpartum depression and children’s receptive and expressive language during the first six years of life: A systematic review of depression timing, status, and chronicity. Developmental Review, 71, 101105. 10.1016/j.dr.2023.101105

[jcv212299-bib-0022] Cox, J. L. , Holden, J. M. , & Sagovsky, R. (1987). Detection of postnatal depression: Development of the 10‐item Edinburgh postnatal depression scale. The British Journal of Psychiatry, 150(6), 782–786. 10.1192/bjp.150.6.782 3651732

[jcv212299-bib-0023] Dagvadorj, A. , Ganbaatar, D. , O. Balogun, O. , Yonemoto, N. , Bavuusuren, B. , Takehara, K. , Mori, R. , & Akahira‐Azuma, M. (2018). Maternal socio‐demographic and psychological predictors for risk of developmental delays among young children in Mongolia. BMC Pediatrics, 18, 1–8. 10.1186/s12887-018-1017-y 29458342 PMC5817794

[jcv212299-bib-0024] DiPietro, J. A. , Novak, M. F. , Costigan, K. A. , Atella, L. D. , & Reusing, S. P. (2006). Maternal psychological distress during pregnancy in relation to child development at age two. Child Development, 77(3), 573–587. 10.1111/j.1467-8624.2006.00891.x 16686789

[jcv212299-bib-0025] Donald, K. A. , Wedderburn, C. J. , Barnett, W. , Nhapi, R. T. , Rehman, A. M. , Stadler, J. A. , Hoffman, N. , Koen, N. , Zar, H. J. , & Stein, D. J. (2019). Risk and protective factors for child development: An observational South African birth cohort. PLoS Medicine, 16(9), e1002920. 10.1371/journal.pmed.1002920 31560687 PMC6764658

[jcv212299-bib-0026] Dubber, S. , Reck, C. , Müller, M. , & Gawlik, S. (2015). Postpartum bonding: The role of perinatal depression, anxiety and maternal–fetal bonding during pregnancy. Archives of Women's Mental Health, 18(2), 187–195. 10.1007/s00737-014-0445-4 25088531

[jcv212299-bib-0027] Dyvik, E. H. (2023). Population density in Sweden in 2022, by county. Statista. Retrieved from https://www.statista.com/statistics/526617/sweden‐population‐density‐by‐county/

[jcv212299-bib-0028] Eriksson, M. , Marschik, P. B. , Tulviste, T. , Almgren, M. , Pérez Pereira, M. , Wehberg, S. , Marjanovič‐Umek, L. , Gayraud, F. , Kovacevic, M. , & Gallego, C. (2012). Differences between girls and boys in emerging language skills: Evidence from 10 language communities. British Journal of Developmental Psychology, 30(2), 326–343. 10.1111/j.2044-835X.2011.02042.x 22550951

[jcv212299-bib-0029] Etchell, A. , Adhikari, A. , Weinberg, L. S. , Choo, A. L. , Garnett, E. O. , Chow, H. M. , & Chang, S.‐E. (2018). A systematic literature review of sex differences in childhood language and brain development. Neuropsychologia, 114, 19–31. 10.1016/j.neuropsychologia.2018.04.011 29654881 PMC5988993

[jcv212299-bib-0030] Fenson, L. , Dale, P. S. , Reznick, J. S. , Bates, E. , Thal, D. J. , Pethick, S. J. , Tomasello, M. , Mervis, C. B. , & Stiles, J. (1994). Variability in early communicative development. Monographs of the Society for Research in Child Development, 59(5), 174–185. 10.2307/1166093 7845413

[jcv212299-bib-0031] Fransson, E. , Sörensen, F. , Kallak, T. K. , Ramklint, M. , Eckerdal, P. , Heimgärtner, M. , Krägeloh‐Mann, I. , & Skalkidou, A. (2020). Maternal perinatal depressive symptoms trajectories and impact on toddler behavior–the importance of symptom duration and maternal bonding. Journal of Affective Disorders, 273, 542–551. 10.1016/j.jad.2020.04.003 32560952

[jcv212299-bib-0032] Friedman, D. D. (2005). 4‐month infant vocal quality in mother‐infant face‐to‐face interaction: 6‐week maternal postpartum depressive symptomatology and infant gender. New York University.

[jcv212299-bib-0033] Galler, J. R. , Harrison, R. H. , Ramsey, F. , Forde, V. , & Butler, S. C. (2000). Maternal depressive symptoms affect infant cognitive development in Barbados. The Journal of Child Psychology and Psychiatry and Allied Disciplines, 41(6), 747–757. 10.1111/1469-7610.00662 11039687

[jcv212299-bib-0034] Garman, E. C. , Cois, A. , Tomlinson, M. , Rotheram‐Borus, M. J. , & Lund, C. (2019). Course of perinatal depressive symptoms among South African women: Associations with child outcomes at 18 and 36 months. Social Psychiatry and Psychiatric Epidemiology, 54(9), 1111–1123. 10.1007/s00127-019-01665-2 30805694

[jcv212299-bib-0035] Gibson, J. L. , Newbury, D. F. , Durkin, K. , Pickles, A. , Conti‐Ramsden, G. , & Toseeb, U. (2021). Pathways from the early language and communication environment to literacy outcomes at the end of primary school; the roles of language development and social development. Oxford Review of Education, 47(2), 260–283. 10.1080/03054985.2020.1824902

[jcv212299-bib-0036] Girard, L.‐C. , Pingault, J.‐B. , Doyle, O. , Falissard, B. , & Tremblay, R. E. (2017). Expressive language and prosocial behaviour in early childhood: Longitudinal associations in the UK Millennium Cohort Study. European Journal of Developmental Psychology, 14(4), 381–398. 10.1080/17405629.2016.1215300

[jcv212299-bib-0037] Griffiths, S. , Suksasilp, C. , Lucas, L. , Sebastian, C. L. , Norbury, C. , & Team, S. (2021). Relationship between early language competence and cognitive emotion regulation in adolescence. Royal Society Open Science, 8(10), 210742. 10.1098/rsos.210742 34754495 PMC8493205

[jcv212299-bib-0038] Havron, N. , Ramus, F. , Heude, B. , Forhan, A. , Cristia, A. , Peyre, H. , Group, E. M.‐C. C. S. , Bernard, J. Y. , Botton, J. , Charles, M. A. , Dargent‐Molina, P. , de Lauzon‐Guillain, B. , Ducimetière, P. , De Agostini, M. , Foliguet, B. , Fritel, X. , Germa, A. , Goua, V. , & Thiebaugeorges, O. (2019). The effect of older siblings on language development as a function of age difference and sex. Psychological Science, 30(9), 1333–1343. 10.1177/0956797619861

[jcv212299-bib-0039] Hay, D. F. , & Kumar, R. (1995). Interpreting the effects of mothers' postnatal depression on children's intelligence: A critique and re‐analysis. Child Psychiatry and Human Development, 25(3), 165–181. 10.1007/BF02251301 7736802

[jcv212299-bib-0040] Hoff, E. (2003). Language development in childhood. In R. M. Lerner , M. A. Easterbrooks , & J. Mistri (Eds.), Handbook of psychology. Developmental psychology (Vol. 6, pp. 171–193). Wiley. 10.1002/0471264385.wei0607

[jcv212299-bib-0041] Hoff, E. (2006). How social contexts support and shape language development. Developmental Review, 26(1), 55–88. 10.1016/j.dr.2005.11.002

[jcv212299-bib-0042] Hoff‐Ginsberg, E. (1998). The relation of birth order and socioeconomic status to children's language experience and language development. Applied PsychoLinguistics, 19(4), 603–629. 10.1017/S0142716400010389

[jcv212299-bib-0043] Hohm, E. , Jennen‐Steinmetz, C. , Schmidt, M. H. , & Laucht, M. (2007). Language development at ten months: Predictive of language outcome and school achievement ten years later? European Child and Adolescent Psychiatry, 16(3), 149–156. 10.1007/s00787-006-0567-y 17347783

[jcv212299-bib-0044] Høivik, M. S. , Burkeland, N. A. , Linaker, O. M. , & Berg‐Nielsen, T. S. (2013). The mother and baby interaction scale: A valid broadband instrument for efficient screening of postpartum interaction? A preliminary validation in a Norwegian community sample. Scandinavian Journal of Caring Sciences, 27(3), 733–739. 10.1111/j.1471-6712.2012.01060.x 22892011

[jcv212299-bib-0045] Hollich, G. J. , Hirsh‐Pasek, K. , Golinkoff, R. M. , Brand, R. J. , Brown, E. , Chung, H. L. , Hennon, E. , Rocroi, C. , & Bloom, L. (2000). Breaking the language barrier: An emergentist coalition model for the origins of word learning. Monographs of the Society for Research in Child Development, 65(3), v–123. 10.1111/1540-5834.00090 12467096

[jcv212299-bib-0046] Huizink, A. C. , & Mulder, E. J. (2006). Maternal smoking, drinking or cannabis use during pregnancy and neurobehavioral and cognitive functioning in human offspring. Neuroscience and Biobehavioral Reviews, 30(1), 24–41. 10.1016/j.neubiorev.2005.04.005 16095697

[jcv212299-bib-0047] Husain, N. , Cruickshank, J. K. , Tomenson, B. , Khan, S. , & Rahman, A. (2012). Maternal depression and infant growth and development in British Pakistani women: A cohort study. BMJ Open, 2(2), e000523. 10.1136/bmjopen-2011-000523 PMC3312074 22436136

[jcv212299-bib-0048] Ibanez, G. , Bernard, J. Y. , Rondet, C. , Peyre, H. , Forhan, A. , Kaminski, M. , Saurel‐Cubizolles, M.‐J. , & Group, E. M.‐C. C. S. (2015). Effects of antenatal maternal depression and anxiety on children’s early cognitive development: A prospective cohort study. PLoS One, 10(8), e0135849. 10.1371/journal.pone.0135849 26317609 PMC4552796

[jcv212299-bib-0049] Junker, D. r. A. , & Stockman, I. J. (2002). Expressive vocabulary of German‐English bilingual toddlers. American Journal of Speech‐Language Pathology, 11(4), 381–394. 10.1044/1058-0360(2002/042

[jcv212299-bib-0050] Kalita, K. N. (2010). Developmental profile of infants born to mothers with postpartum depression and anxiety: A comparative study. Journal of Indian Association for Child and Adolescent Mental Health, 6(1), 3–12. 10.1177/0973134220100102

[jcv212299-bib-0051] Kaplan, P. S. , Danko, C. M. , Everhart, K. D. , Diaz, A. , Asherin, R. M. , Vogeli, J. M. , & Fekri, S. M. (2014). Maternal depression and expressive communication in one‐year‐old infants. Infant Behavior and Development, 37(3), 398–405. 10.1016/j.infbeh.2014.05.008 24953222 PMC4106459

[jcv212299-bib-0052] Kaplan, P. S. , Danko, C. M. , Kalinka, C. J. , & Cejka, A. M. (2012). A developmental decline in the learning‐promoting effects of infant‐directed speech for infants of mothers with chronically elevated symptoms of depression. Infant Behavior and Development, 35(3), 369–379. 10.1016/j.infbeh.2012.02.009 22721737 PMC3914005

[jcv212299-bib-0053] Keim, S. A. , Daniels, J. L. , Dole, N. , Herring, A. H. , Siega‐Riz, A. M. , & Scheidt, P. C. (2011). A prospective study of maternal anxiety, perceived stress, and depressive symptoms in relation to infant cognitive development. Early Human Development, 87(5), 373–380. 10.1016/j.earlhumdev.2011.02.004 21420261 PMC3079050

[jcv212299-bib-0054] Koutra, K. , Chatzi, L. , Bagkeris, M. , Vassilaki, M. , Bitsios, P. , & Kogevinas, M. (2013). Antenatal and postnatal maternal mental health as determinants of infant neurodevelopment at 18 months of age in a mother–child cohort (Rhea Study) in Crete, Greece. Social Psychiatry and Psychiatric Epidemiology, 48(8), 1335–1345. 10.1007/s00127-012-0636-0 23248031

[jcv212299-bib-0055] Kovas, Y. , Hayiou‐Thomas, M. E. , Oliver, B. , Dale, P. S. , Bishop, D. V. , & Plomin, R. (2005). Genetic influences in different aspects of language development: The etiology of language skills in 4.5‐year‐old twins. Child Development, 76(3), 632–651. 10.1111/j.1467-8624.2005.00868.x 15892783

[jcv212299-bib-0056] Kuhl, P. K. (2007). Is speech learning ‘gated’by the social brain? Developmental Science, 10(1), 110–120. 10.1111/j.1467-7687.2007.00572.x 17181708

[jcv212299-bib-0057] Kurstjens, S. , & Wolke, D. (2001). Effects of maternal depression on cognitive development of children over the first 7 years of life. Journal of Child Psychology and Psychiatry, 42(5), 623–636. 10.1111/1469-7610.00758 11464967

[jcv212299-bib-0058] Lam‐Cassettari, C. , & Kohlhoff, J. (2020). Effect of maternal depression on infant‐directed speech to prelinguistic infants: Implications for language development. PLoS One, 15(7), e0236787. 10.1371/journal.pone.0236787 32730322 PMC7392317

[jcv212299-bib-0059] Le Bas, G. A. , Youssef, G. J. , Macdonald, J. A. , Rossen, L. , Teague, S. J. , Kothe, E. J. , McIntosh, J. E. , Olsson, C. A. , & Hutchinson, D. M. (2020). The role of antenatal and postnatal maternal bonding in infant development: A systematic review and meta‐analysis. Social Development, 29(1), 3–20. 10.1111/sode.12392

[jcv212299-bib-0060] Liberman, Z. , Woodward, A. L. , Keysar, B. , & Kinzler, K. D. (2017). Exposure to multiple languages enhances communication skills in infancy. Developmental Science, 20(1), e12420. 10.1111/desc.12420 PMC5031504 27002779

[jcv212299-bib-0061] Lin, Y. , Xu, J. , Huang, J. , Jia, Y. , Zhang, J. , Yan, C. , & Zhang, J. (2017). Effects of prenatal and postnatal maternal emotional stress on toddlers’ cognitive and temperamental development. Journal of Affective Disorders, 207, 9–17. 10.1016/j.jad.2016.09.010 27665073

[jcv212299-bib-0062] MacGinty, R. , Kariuki, S. , Barnett, W. , Wedderburn, C. , Hardy, A. , Hoffman, N. , Newton, C. , Zar, H. , Donald, K. , & Stein, D. (2020). Associations of antenatal maternal psychological distress with infant birth and development outcomes: Results from a South African birth cohort. Comprehensive Psychiatry, 96, 152128. 10.1016/j.comppsych.2019.152128 31715335 PMC6945113

[jcv212299-bib-0063] Malin, J. L. , Karberg, E. , Cabrera, N. J. , Rowe, M. , Cristaforo, T. , & Tamis‐LeMonda, C. S. (2012). Father–toddler communication in low‐income families: The role of paternal education and depressive symptoms. Family Science, 3(3–4), 155–163. 10.1080/19424620.2012.779423 25232446 PMC4163955

[jcv212299-bib-0064] Mitchell, A. R. , Gordon, H. , Lindquist, A. , Walker, S. P. , Homer, C. S. , Middleton, A. , Cluver, C. A. , Tong, S. , & Hastie, R. (2023). Prevalence of perinatal depression in low‐and middle‐income countries: A systematic review and meta‐analysis. JAMA Psychiatry, 80(5), 425–431. 10.1001/jamapsychiatry.2023.0069 36884232 PMC9996459

[jcv212299-bib-0065] Moe, V. , Fredriksen, E. , Kjellevold, M. , Dahl, L. , Markhus, M. W. , Stormark, K. M. , von Soest, T. , Olafsen, K. S. , Vannebo, U. T. , & Smith, L. (2019). Little in Norway: A prospective longitudinal community‐based cohort from pregnancy to child age 18 months. BMJ Open, 9(12), e031050. 10.1136/bmjopen-2019-031050 PMC6955541 31892648

[jcv212299-bib-0066] Moehler, E. , Brunner, R. , Wiebel, A. , Reck, C. , & Resch, F. (2006). Maternal depressive symptoms in the postnatal period are associated with long‐term impairment of mother–child bonding. Archives of Women's Mental Health, 9(5), 273–278. 10.1007/s00737-006-0149-5 16937313

[jcv212299-bib-0067] Mughal, M. K. , Giallo, R. , Arnold, P. , Benzies, K. , Kehler, H. , Bright, K. , & Kingston, D. (2018). Trajectories of maternal stress and anxiety from pregnancy to three years and child development at 3 years of age: Findings from the All Our Families (AOF) pregnancy cohort. Journal of Affective Disorders, 234, 318–326. 10.1016/j.jad.2018.02.095 29604550

[jcv212299-bib-0068] Mughal, M. K. , Giallo, R. , Arnold, P. D. , Kehler, H. , Bright, K. , Benzies, K. , Wajid, A. , & Kingston, D. (2019). Trajectories of maternal distress and risk of child developmental delays: Findings from the All Our Families (AOF) pregnancy cohort. Journal of Affective Disorders, 248, 1–12. 10.1016/j.jad.2018.12.132 30690110

[jcv212299-bib-0069] Muñez, D. , Bull, R. , & Lee, K. (2022). Maternal education and siblings: Agents of cognitive development in kindergarten. Developmental Science, 25(4), e13218. 10.1111/desc.13218 34964196

[jcv212299-bib-0070] Muñoz‐Rocha, T. V. , y Ortiz, M. T. , Romero, M. , Pantic, I. , Schnaas, L. , Bellinger, D. , Claus‐Henn, B. , Wright, R. , Wright, R. O. , & Téllez‐Rojo, M. M. (2018). Prenatal co‐exposure to manganese and depression and 24‐months neurodevelopment. Neurotoxicology, 64, 134–141. 10.1016/j.neuro.2017.07.007 28728787 PMC5771973

[jcv212299-bib-0071] Murray, L. , Cooper, P. , Arteche, A. , Stein, A. , & Tomlinson, M. (2016). Randomized controlled trial of a home‐visiting intervention on infant cognitive development in peri‐urban South Africa. Developmental Medicine and Child Neurology, 58(3), 270–276. 10.1111/dmcn.12873 26303135 PMC4945696

[jcv212299-bib-0072] Netsi, E. , Pearson, R. , Murray, L. , Cooper, P. , Craske, M. , & Stein, A. (2018). Association of persistent and severe postnatal depression with child outcomes. JAMA Psychiatry, 75(3), 247–253. 10.1001/jamapsychiatry.2017.4363 29387878 PMC5885957

[jcv212299-bib-0073] Nulman, I. , Koren, G. , Rovet, J. , Barrera, M. , Pulver, A. , Streiner, D. , & Feldman, B. (2012). Neurodevelopment of children following prenatal exposure to venlafaxine, selective serotonin reuptake inhibitors, or untreated maternal depression. American Journal of Psychiatry, 169(11), 1165–1174. 10.1176/appi.ajp.2012.11111721 23128923

[jcv212299-bib-0074] Ohara, M. , Okada, T. , Kubota, C. , Nakamura, Y. , Shiino, T. , Aleksic, B. , Morikawa, M. , Yamauchi, A. , Uno, Y. , Murase, S. , Goto, S. , Kanai, A. , Masuda, T. , Ando, M. , & Ozaki, N. (2017). Relationship between maternal depression and bonding failure: A prospective cohort study of pregnant women. Psychiatry and Clinical Neurosciences, 71(10), 733–741. 10.1111/pcn.12541 28556440

[jcv212299-bib-0075] O’Higgins, M. , Roberts, I. S. J. , Glover, V. , & Taylor, A. (2013). Mother‐child bonding at 1 year; associations with symptoms of postnatal depression and bonding in the first few weeks. Archives of Women's Mental Health, 16(5), 381–389. 10.1007/s00737-013-0354-y 23604546

[jcv212299-bib-0076] O'Leary, N. , Jairaj, C. , Molloy, E. J. , McAuliffe, F. M. , Nixon, E. , & O'Keane, V. (2019). Antenatal depression and the impact on infant cognitive, language and motor development at six and twelve months postpartum. Early Human Development, 134, 41–46. 10.1016/j.earlhumdev.2019.05.021 31176100

[jcv212299-bib-0077] Osborne, S. , Biaggi, A. , Chua, T. , Du Preez, A. , Hazelgrove, K. , Nikkheslat, N. , Previti, G. , Zunszain, P. , Conroy, S. , & Pariante, C. (2018). Antenatal depression programs cortisol stress reactivity in offspring through increased maternal inflammation and cortisol in pregnancy: The psychiatry research and motherhood–depression (PRAM‐D) study. Psychoneuroendocrinology, 98, 211–221. 10.1016/j.psyneuen.2018.06.017 30033161 PMC6215770

[jcv212299-bib-0078] Özçalışkan, Ş. , & Goldin‐Meadow, S. (2010). Sex differences in language first appear in gesture. Developmental Science, 13(5), 752–760. 10.1111/j.1467-7687.2009.00933.x 20712741 PMC2923747

[jcv212299-bib-0079] Pace, A. , Luo, R. , Hirsh‐Pasek, K. , & Golinkoff, R. M. (2017). Identifying pathways between socioeconomic status and language development. Annual Review of Linguistics, 3(1), 285–308. 10.1146/annurev-linguistics-011516-034226

[jcv212299-bib-0080] Parfitt, Y. , Pike, A. , & Ayers, S. (2014). Infant developmental outcomes: A family systems perspective. Infant and Child Development, 23(4), 353–373. 10.1002/icd.1830

[jcv212299-bib-0081] Paulson, J. F. , Keefe, H. A. , & Leiferman, J. A. (2009). Early parental depression and child language development. Journal of Child Psychology and Psychiatry, 50(3), 254–262. 10.1111/j.1469-7610.2008.01973.x 19175819

[jcv212299-bib-0082] Piteo, A. M. , Yelland, L. N. , & Makrides, M. (2012). Does maternal depression predict developmental outcome in 18 month old infants? Early Human Development, 88(8), 651–655. 10.1016/j.earlhumdev.2012.01.013 22361258

[jcv212299-bib-0083] Pop, V. J. , de Vries, E. , van Baar, A. L. , Waelkens, J. , De Rooy, H. , Horsten, M. , Donkers, M. , Komproe, I. , Van Son, M. , & Vader, H. (1995). Maternal thyroid peroxidase antibodies during pregnancy: A marker of impaired child development? Journal of Clinical Endocrinology and Metabolism, 80(12), 3561–3566. 10.1210/jcem.80.12.8530599 8530599

[jcv212299-bib-0084] Punamäki, R.‐L. , Diab, S. Y. , Isosävi, S. , Kuittinen, S. , & Qouta, S. R. (2018). Maternal pre‐and postnatal mental health and infant development in war conditions: The Gaza Infant Study. Psychological Trauma: Theory, Research, Practice, and Policy, 10(2), 144–153. 10.1037/tra0000275 28406660

[jcv212299-bib-0085] Quevedo, L. , Silva, R. , Godoy, R. , Jansen, K. , Matos, M. , Tavares Pinheiro, K. , & Pinheiro, R. (2012). The impact of maternal post‐partum depression on the language development of children at 12 months. Child: Care, Health and Development, 38(3), 420–424. 10.1111/j.1365-2214.2011.01251.x 21651606

[jcv212299-bib-0086] Ramakrishna, S. , Cooklin, A. R. , & Leach, L. S. (2019). Comorbid anxiety and depression: A community‐based study examining symptomology and correlates during the postpartum period. Journal of Reproductive and Infant Psychology, 37(5), 468–479. 10.1080/02646838.2019.1578870 30786765

[jcv212299-bib-0087] Rescorla, L. , & Alley, A. (2001). Validation of the Language Development Survey (LDS): A parent report tool for identifying language delay in toddlers. Journal of Speech, Language, and Hearing Research, 44(2), 434–445. 10.1044/1092-4388(2001/035 11324663

[jcv212299-bib-0088] Rinaldi, P. , Pasqualetti, P. , Volterra, V. , & Caselli, M. C. (2023). Gender differences in early stages of language development. Some evidence and possible explanations. Journal of Neuroscience Research, 101(5), 643–653. 10.1002/jnr.24914 34240751

[jcv212299-bib-0089] Rogers, A. , Obst, S. , Teague, S. J. , Rossen, L. , Spry, E. A. , Macdonald, J. A. , Sunderland, M. , Olsson, C. A. , Youssef, G. , & Hutchinson, D. (2020). Association between maternal perinatal depression and anxiety and child and adolescent development: A meta‐analysis. JAMA Pediatrics, 174(11), 1082–1092. 10.1001/jamapediatrics.2020.2910 32926075 PMC7490743

[jcv212299-bib-0090] Rossen, L. , Hutchinson, D. , Wilson, J. , Burns, L. , A Olsson, C. , Allsop, S. , J Elliott, E. , Jacobs, S. , Macdonald, J. A. , & Mattick, R. P. (2016). Predictors of postnatal mother‐infant bonding: The role of antenatal bonding, maternal substance use and mental health. Archives of Women's Mental Health, 19(4), 609–622. 10.1007/s00737-016-0602-z 26867547

[jcv212299-bib-0091] Rotheram‐Fuller, E. J. , Tomlinson, M. , Scheffler, A. , Weichle, T. W. , Hayati Rezvan, P. , Comulada, W. S. , & Rotheram‐Borus, M. J. (2018). Maternal patterns of antenatal and postnatal depressed mood and the impact on child health at 3‐years postpartum. Journal of Consulting and Clinical Psychology, 86(3), 218–230. 10.1037/ccp0000281 29504791 PMC5842813

[jcv212299-bib-0092] Rowe, M. L. , & Weisleder, A. (2020). Language development in context. Annual Review of Developmental Psychology, 2(1), 201–223. 10.1146/annurev-devpsych-042220-121816

[jcv212299-bib-0093] Sansavini, A. , Favilla, M. E. , Guasti, M. T. , Marini, A. , Millepiedi, S. , Di Martino, M. V. , Vecchi, S. , Battajon, N. , Bertolo, L. , Capirci, O. , Carretti, B. , Colatei, M. P. , Frioni, C. , Marotta, L. , Massa, S. , Michelazzo, L. , Pecini, C. , Piazzalunga, S. , Pieretti, M. , …, & Lorusso, M. L. (2021). Developmental language disorder: Early predictors, age for the diagnosis, and diagnostic tools. A scoping review. Brain Sciences, 11(5), 654. 10.3390/brainsci11050654 34067874 PMC8156743

[jcv212299-bib-0094] Schaadt, G. , Zsido, R. G. , Villringer, A. , Obrig, H. , Männel, C. , & Sacher, J. (2022). Association of postpartum maternal mood with infant speech perception at 2 and 6.5 Months of age. JAMA Network Open, 5(9), e2232672. 10.1001/jamanetworkopen.2022.32672 36129707 PMC9494190

[jcv212299-bib-0095] Schjølberg, S. , Eadie, P. , Zachrisson, H. D. , Øyen, A.‐S. , & Prior, M. (2011). Predicting language development at age 18 months: Data from the Norwegian mother and child cohort study. Journal of Developmental and Behavioral Pediatrics, 32(5), 375–383. 10.1097/DBP.0b013e31821bd1dd 21546853

[jcv212299-bib-0096] Seay, D. M. (2019). Longitudinal relations among adolescent mothers' depression, negative parenting, social support and young children's developmental outcomes. Doctoral Thesis. Arizona State University.

[jcv212299-bib-0097] Senturk, V. (2017). Changing family relationships, perinatal depression, and child development in Turkey. Doctoral Thesis. King's College London.

[jcv212299-bib-0098] Sim, F. , Thompson, L. , Marryat, L. , Ramparsad, N. , & Wilson, P. (2019). Predictive validity of preschool screening tools for language and behavioural difficulties: A PRISMA systematic review. PLoS One, 14(2), e0211409. 10.1371/journal.pone.0211409 30716083 PMC6361441

[jcv212299-bib-0099] Slomian, J. , Honvo, G. , Emonts, P. , Reginster, J.‐Y. , & Bruyère, O. (2019). Consequences of maternal postpartum depression: A systematic review of maternal and infant outcomes. Women's Health, 15, 1745506519844044. 10.1177/1745506519844044 PMC6492376 31035856

[jcv212299-bib-0100] Snijders, V. E. , Bogicevic, L. , Verhoeven, M. , & van Baar, A. L. (2020). Toddlers’ language development: The gradual effect of gestational age, attention capacities, and maternal sensitivity. International Journal of Environmental Research and Public Health, 17(21), 7926. 10.3390/ijerph17217926 33137895 PMC7663656

[jcv212299-bib-0101] Stein, A. , Pearson, R. M. , Goodman, S. H. , Rapa, E. , Rahman, A. , McCallum, M. , Howard, L. M. , & Pariante, C. M. (2014). Effects of perinatal mental disorders on the fetus and child. The Lancet, 384(9956), 1800–1819. 10.1016/S0140-6736(14)61277-0 25455250

[jcv212299-bib-0102] Stow, C. , & Dodd, B. (2003). Providing an equitable service to bilingual children in the UK: A review. International Journal of Language and Communication Disorders, 38(4), 351–377. 10.1080/1368282031000156888 14578052

[jcv212299-bib-0103] Sutherland, S. , & Brunwasser, S. M. (2018). Sex differences in vulnerability to prenatal stress: A review of the recent literature. Current Psychiatry Reports, 20(11), 1–12. 10.1007/s11920-018-0961-4 30229468 PMC6329286

[jcv212299-bib-0104] Tenenbaum, E. J. , Sobel, D. M. , Sheinkopf, S. J. , Malle, B. F. , & Morgan, J. L. (2015). Attention to the mouth and gaze following in infancy predict language development. Journal of Child Language, 42(6), 1173–1190. 10.1017/S0305000914000725 25403090 PMC8281329

[jcv212299-bib-0105] Thorpe, K. (2006). Twin children's language development. Early Human Development, 82(6), 387–395. 10.1016/j.earlhumdev.2006.03.012 16690234

[jcv212299-bib-0106] Tichelman, E. , Westerneng, M. , Witteveen, A. B. , Van Baar, A. L. , Van Der Horst, H. E. , De Jonge, A. , Berger, M. Y. , Schellevis, F. G. , Burger, H. , & Peters, L. L. (2019). Correlates of prenatal and postnatal mother‐to‐infant bonding quality: A systematic review. PLoS One, 14(9), e0222998. 10.1371/journal.pone.0222998 31550274 PMC6759162

[jcv212299-bib-0107] Tronick, E. , & Reck, C. (2009). Infants of depressed mothers. Harvard Review of Psychiatry, 17(2), 147–156. 10.1080/10673220902899714 19373622

[jcv212299-bib-0108] Tse, A. C. , Rich‐Edwards, J. W. , Rifas‐Shiman, S. L. , Gillman, M. W. , & Oken, E. (2010). Association of maternal prenatal depressive symptoms with child cognition at age 3 years. Paediatric & Perinatal Epidemiology, 24(3), 232–240. 10.1111/j.1365-3016.2010.01113.x 20415752 PMC2860615

[jcv212299-bib-0109] Tu, H.‐F. , Fransson, E. , Kallak, T. K. , Elofsson, U. , Ramklint, M. , & Skalkidou, A. (2023). Cohort profile: The U‐BIRTH study on peripartum depression and child development in Sweden. BMJ Open, 13(11), e072839. 10.1136/bmjopen-2023-072839 PMC10649626 37949626

[jcv212299-bib-0110] Verhoef, E. , Shapland, C. Y. , Fisher, S. E. , Dale, P. S. , & St Pourcain, B. (2021). The developmental origins of genetic factors influencing language and literacy: Associations with early‐childhood vocabulary. Journal of Child Psychology and Psychiatry, 62(6), 728–738. 10.1111/jcpp.13399 32924135

[jcv212299-bib-0111] Weikum, W. M. , Oberlander, T. F. , Hensch, T. K. , & Werker, J. F. (2012). Prenatal exposure to antidepressants and depressed maternal mood alter trajectory of infant speech perception. Proceedings of the National Academy of Sciences, 109(supplement_2), 17221–17227. 10.1073/pnas.1121263109 PMC3477387 23045665

[jcv212299-bib-0112] Wickberg, B. , & Hwang, C. (1996). The Edinburgh postnatal depression scale: Validation on a Swedish community sample. Acta Psychiatrica Scandinavica, 94(3), 181–184. 10.1111/j.1600-0447.1996.tb09845.x 8891084

[jcv212299-bib-0113] Woody, C. , Ferrari, A. , Siskind, D. , Whiteford, H. , & Harris, M. (2017). A systematic review and meta‐regression of the prevalence and incidence of perinatal depression. Journal of Affective Disorders, 219, 86–92. 10.1016/j.jad.2017.05.003 28531848

