# Supplementary material for: Do the early social environment and persistent peripartum depressive symptoms shape toddlers' expressive language?
Source: JCPP Adv. 2025 Feb 11;5(3):e12299. doi: 10.1002/jcv2.12299 (PMC12446726; doi:10.1002/jcv2.12299)
Supplement: Supplementary file 1 — Supporting Information S1 [file JCV2-5-e12299-s001.docx]

**Supporting Information**


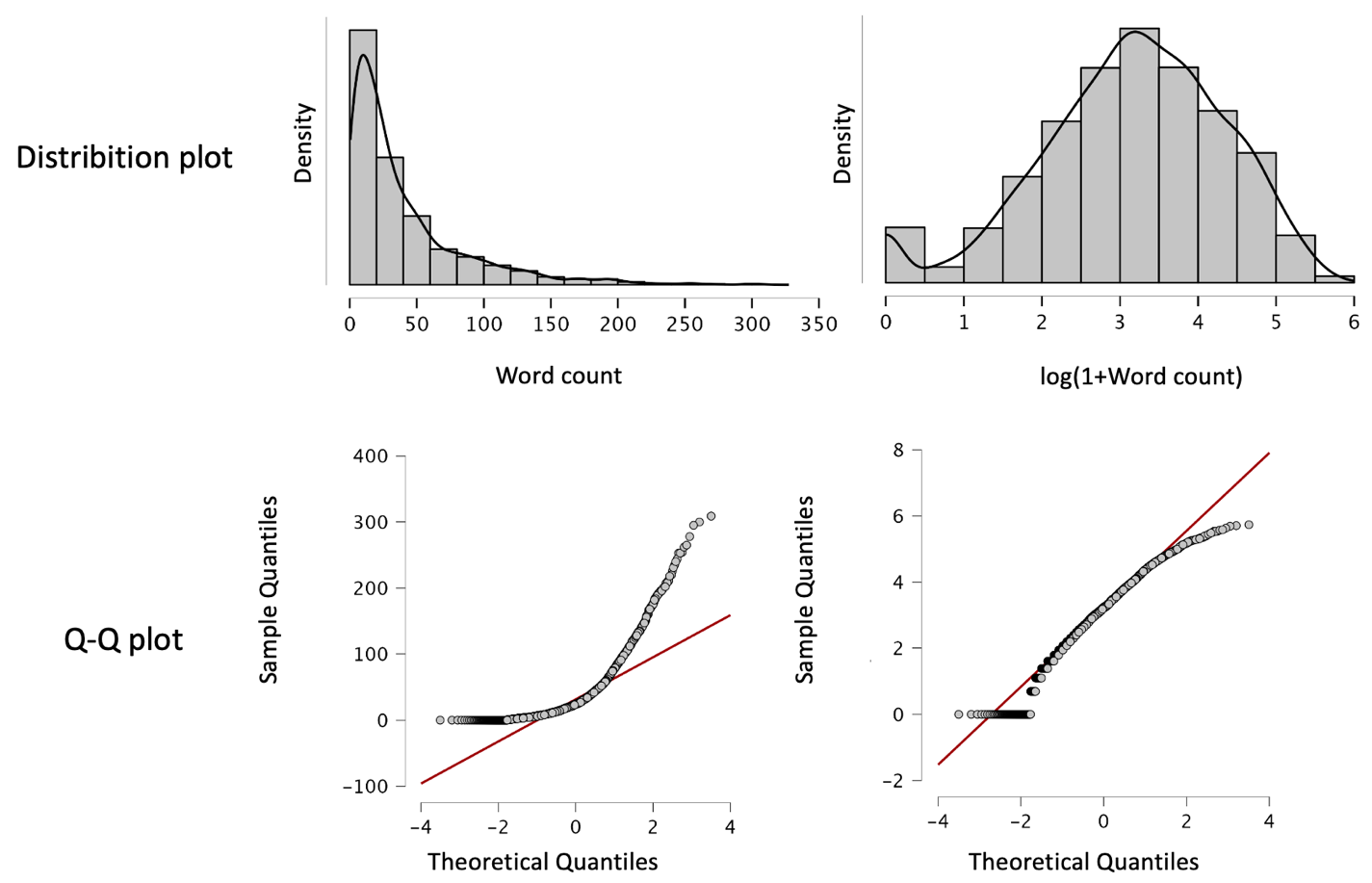


**Figure S1.** The upper left panel shows the distribution of language word counts (raw scores) with its corresponding Q-Q plot below it. On the right side, the distribution and Q-Q plots are presented for the logarithm-transformed word counts. Based on the visual inspection, the logarithm-transformed value is used as an outcome value in the multivariable linear regression analyses.


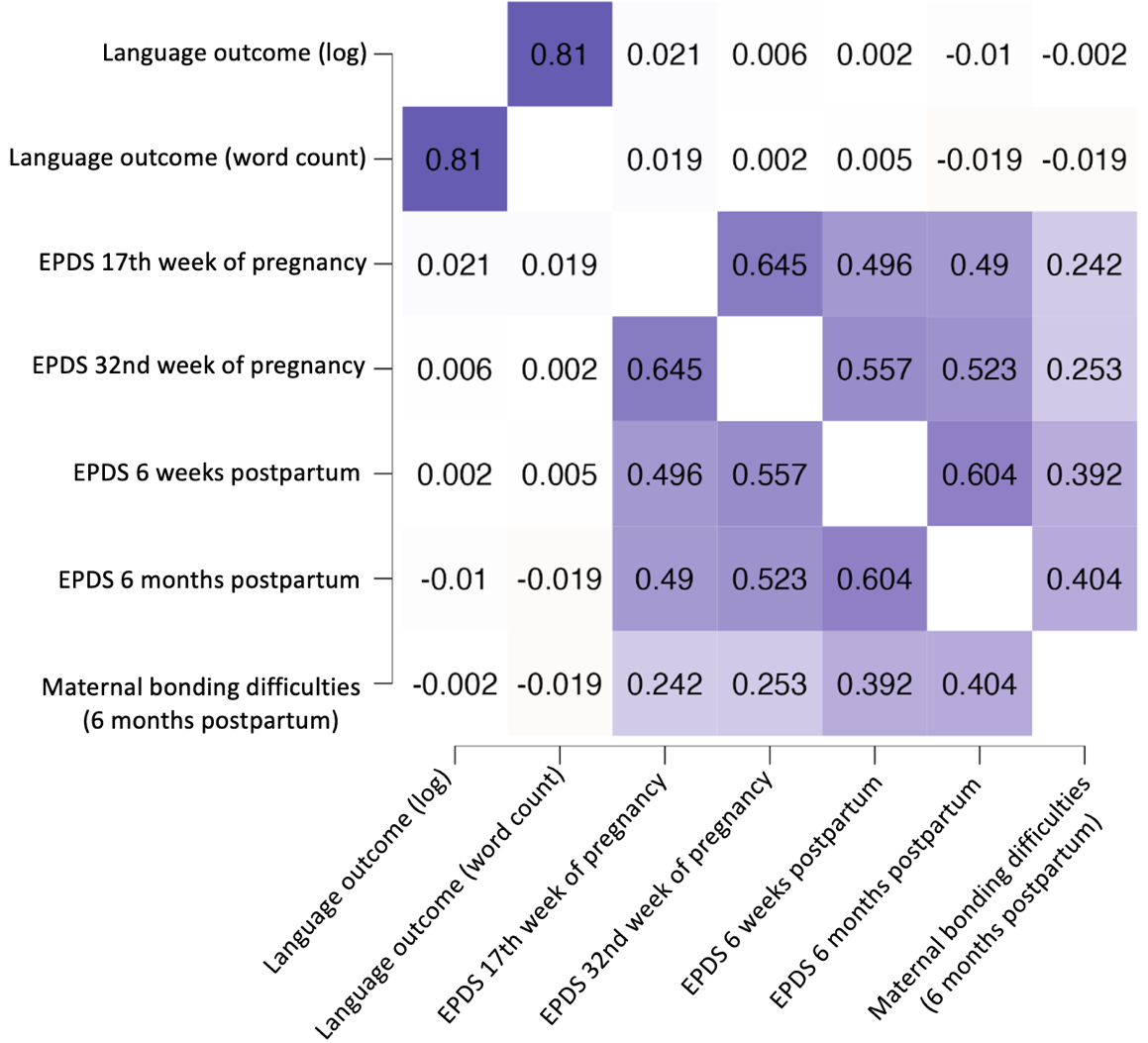


**Figure S2.** Heatmap of Pearson’s correlations among language outcome, depressive symotpms across four time points, and maternal bonding difficulties. The heatmap uses a purple gradient to represent the strength of the siginificant Pearson's correlation coefficients. White indicates non-significant correlations, while darker shades of purple indicate higher correlation coefficients. The numerical values in the figure represent the actual correlation coefficients (r).EPDS: the Edinburgh Postnatal Depression Scale.

**Table S1.** Summary of multivariable linear regression of perinatal depressive symptoms (4 time points) and toddler expressive language development (Model 1 and Model 2)

| Variable | Model 1 | | | Model 2 | | |
| --- | --- | --- | --- | --- | --- | --- |
|  | F(4, 2171) = 0.543, p = 0.704, R^2^ = 0.001 | | | **F(9, 1779) = 1.762, p = 0.071, R^2^ = 0.009** | | |
|  | β | 95% CI | VIF | β | 95% CI | VIF |
| EPDS 17th week of pregnancy | 0.010 | [-0.006, 0.025] | 1.843 | 0.016 | [-3.6x10^-5^, 0.032] | 1.352 |
| EPDS 32nd week of pregnancy | -0.001 | [-0.018, 0.015] | 2.023 | -0.005 | [-0.022, 0.013] | 1.428 |
| EPDS 6 weeks postpartum | 6.5x10^-4^ | [-0.014, 0.016] | 1.832 | -0.003 | [-0.018, 0.013] | 1.364 |
| EPDS 6 months postpartum | -0.007 | [-0.021, 0.008] | 1.754 | -0.001 | [-0.017, 0.014] | 1.341 |
| Maternal bonding difficulties (6 months postpartum) |  |  |  | -0.002 | [-0.010, 0.006] | 1.119 |
| Birth order (First born with other siblings) |  |  |  | 0.094 | [-0.043, 0.231] | 1.005 |
| **Birth order (Second born)** |  |  |  | **-0.167*** | **[-0.308, -0.026]** |  |
| Birth order (Third or fourth born) |  |  |  | -0.358 | [-0.824, 0.108] |  |
| Multilingual household (Yes) |  |  |  | -0.027 | [-0.168, 0.113] | 1.004 |
| **Sex (Girl)** |  |  |  |  |  |  |
| Preterm birth (Yes) |  |  |  |  |  |  |
| Admission to NICU at birth (Yes) |  |  |  |  |  |  |
| Twin birth (Yes) |  |  |  |  |  |  |
| **Family member with delayed talking (Yes)** |  |  |  |  |  |  |
| **Living area (small urban area)** |  |  |  |  |  |  |
| **Living area (countryside)** |  |  |  |  |  |  |
| **Mother’s age at partus** |  |  |  |  |  |  |
| **Pregnancy length** |  |  |  |  |  |  |
| Mother’s education (university) |  |  |  |  |  |  |
| Mother’s employment (full-time) |  |  |  |  |  |  |
| Mother’s employment (part-time or on leave) |  |  |  |  |  |  |
| Mother’s history of smoking (Yes) |  |  |  |  |  |  |
|  |  |  |  |  |  |  |
| Regression formular | Model 1_(H0)_ =3.138 + 0.010(EPDS 17th week of pregnancy) − 0.001(EPDS 32nd week of pregnancy) + 6.491×10^−4^(EPDS 6 weeks postpartum) − 0.007(EPDS 6 months postpartum) | | | Model 2_(H0)​_ = 3.222 + 0.016(EPDS 17th week of pregnancy) − 0.005(EPDS 32nd week of pregnancy) – 0.003(EPDS 6 weeks postpartum) – 0.001(EPDS 6 months postpartum) − 0.002(Maternal bonding difficulties) + 0.094(Birth order, First) − 0.167(Birth order, Second) − 0.358(Birth order, Third and fourth) − 0.027(Multilingual household,Yes) | | |
| * p < .05. β is unstandardized. Bold text denotes statistically significant results. Birth order: firstborn and single child is the reference. Living area: big city is the reference. Abbreviations: EPDS, The Edinburgh postnatal depression scale; NICU, neonatal intensive care unit; CI, confidence interval; VIF, variance inflation factor. | | | | | | |

**Table S2.** Group difference in language (word count) based on different catetorical variables.

| **Categorical variable** | **Statistical method** | **Key results** |
| --- | --- | --- |
| Sex | Independent t-test | t(2174) = -7.850, p < 0.001, Cohen’s d = -0.337   - Boy: n = 1122, mean = 33.2, SD = 38.5, SE = 1.1, coefficient of variation = 1.2 - Girl: n = 1054, mean = 48.2, SD = 50.1, SE = 1.5, coefficient of variation = 1.0 |
| Family history with late talkers | Independent t-test | t(2026) = 3.578, p < 0.001, Cohen’s d = 0.236   - No: n = 1765, mean = 43.1, SD = 46.6, SE = 1.1, coefficient of variation = 1.1 - Yesl: n = 263, mean = 32.4, SD = 35.3, SE = 2.2, coefficient of variation = 1.1 |
| Birth order | ANOVA | F(3, 2172) = 3.295, p = 0.020   - Firstborn and only child: n = 1346, mean = 41.4, SD = 46.0, SE = 1.3, coefficient of variable = 1.1 - Firstborn with siblings: n = 400, mean = 43.1, SD = 45.3, SE = 2.3, coefficient of variable = 1.1 - Second born: n = 400, mean = 35.5, SD = 41.6, SE = 2.1, coefficient of variable = 1.2 - Third and fourth born: n = 30, mean = 29.6, SD = 40.9, SE = 7.5, coefficient of variable = 1.4 |
| Living area | ANOVA | F(2, 2069) = 7.601, p < 0.001   - A big city: n = 1021, mean = 44.4, SD = 46.7, SE = 1.5, coefficient of variable = 1.1 - A small urben area: n = 558, mean = 39.3, SD = 46.0, SE = 1.9, coefficient of variable = 1.2 - Countryside: n = 493, mean = 35.0, SD = 40.0, SE = 1.8, coefficient of variable = 1.1 |
| ANOVA: alanysis of variance; SD: standard deviation; SE: standard error  Note: the birth order was registered in the cohort data at birth. For firstborns with siblings, these siblings were most likely older than the firstborn child. In very few cases, they were twins. | | |

**Table S3.** Summary of multivariable linear regression of prenatal depressive symptoms (average score) and toddler expressive language development

| Variable | Model 1 | | Model 2 | | | Model 3 | | |  |
| --- | --- | --- | --- | --- | --- | --- | --- | --- | --- |
|  | F(1, 1968) = 0.276, p = 0.6, R^2^ = 0 | | **F(6, 1633) = 2.436, p = 0.024, R^2^ = 0.009** | | | **F(19, 1528) = 7.319, p < .001, R^2^ = 0.083** | | |  |
|  | β | 95% CI | β | 95% CI | VIF | β | 95% CI | VIF |  |
| Mean of EPDS score during pregnancy | 0.004 | [-0.010, 0.017] | 0.010 | [-0.004, 0.025] | 1.048 | 0.009 | [-0.006, 0.024] | 1.081 |  |
| Maternal bonding difficulties (6 months postpartum) |  |  | -0.004 | [-0.011, 0.006] | 1.045 | -0.003 | [-0.012, 0.005] | 1.055 |  |
| Birth order (First born with other siblings) |  |  | 0.095 | [-0.049, 0.239] | 1.002 | 0.024 | [-0.122, 0.170] | 1.023 |  |
| **Birth order (Second born)** |  |  | **-0.200**** | **[-0.347, -0.053]** |  | **-0.172**** | **[-0.320, -0.024]** |  |  |
| Birth order (Third or fourth born) |  |  | -0.177 | [-0.776, 0.221] |  | -0.200 | [-0.699, 0.299] |  |  |
| Multilingual household (Yes) |  |  | -0.020 | [-0.166, 0.127] | 1.003 | -0.073 | [-0.221, 0.075] | 1.022 |  |
| **Sex (Girl)** |  |  |  |  |  | **0.398***** | **[0.289, 0.507]** | **1.010** |  |
| Preterm birth (Yes) |  |  |  |  |  | 0.736 | [-0.136, 1.607] | 1.084 |  |
| Admission to NICU at birth (Yes) |  |  |  |  |  | -0.064 | [-0.255, 0.126] | 1.063 |  |
| Twin birth (Yes) |  |  |  |  |  | -0.286 | [-0.636, 0.064] | 1.042 |  |
| **Family member with delayed talking (Yes)** |  |  |  |  |  | **-0.181*** | **[-0.346, -0.015]** | **1.014** |  |
| **Living area (urban)** |  |  |  |  |  | **-0.223***** | **[-0.356, -0.091]** | **1.019** |  |
| **Living area (countryside)** |  |  |  |  |  | **-0.294***** | **[-0.431, -0.156]** |  |  |
| **Mother’s age at partus** |  |  |  |  |  | **-0.023***** | **[-0.036, -0.010]** | **1.050** |  |
| **Pregnancy length** |  |  |  |  |  | **0.006***** | **[0.005, 0.006]** | **1.138** |  |
| Mother’s education (university) |  |  |  |  |  | 0.123 | [-0.038, 0.284] | 1.083 |  |
| Mother’s employment (full-time) |  |  |  |  |  | -0.058 | [-0.336, 0.220] | 1.031 |  |
| Mother’s employment (part-time or on leave) |  |  |  |  |  | -0.038 | [-0.324, 0.249] |  |  |
| Mother’s history of smoking (Yes) |  |  |  |  |  | 0.119 | [-0.007, 0.245] | 1.041 |  |
| * p < .05, ** p < .01, *** p < .001. β is unstandardized. Bold text denotes statistically significant results.  Birth order: reference, firstborn and single child is the reference. Living area: big city is the reference.  Abbreviations: EPDS, The Edinburgh postnatal depression scale; NICU, neonatal intensive care unit; CI, confidence interval; VIF, variance inflation factor. | | | | | | | | | |

**Table S4.** Summary of multivariable linear regression of postnatal depressive symptoms (average score) and toddler expressive language development

| Variable | Model 1 | | Model 2 | | | Model 3 | | |
| --- | --- | --- | --- | --- | --- | --- | --- | --- |
|  | F(1, 1905) = 9.3x10^-4^, p = 0.976, R^2^ = 0 | | **F(6, 1689) = 2.173, p = 0.043, R^2^ = 0.008** | | | **F(19, 1500) = 7.962, p < .001, R^2^ = 0.079** | | |
|  | β | 95% CI | β | 95% CI | VIF | β | 95% CI | VIF |
| Mean of EPDS score postpartum | -2.0x10^-4^ | [-0.013, 0.013] | 0.004 | [-0.011, 0.018] | 1.130 | 0.004 | [-0.012, 0.019] | 1.157 |
| Maternal bonding difficulties (6 months postpartum) |  |  | -0.003 | [-0.012, 0.006] | 1.125 | -0.004 | [-0.013, 0.006] | 1.140 |
| Birth order (First born with other siblings) |  |  | 0.129 | [-0.011, 0.270] | 1.003 | 0.074 | [-0.075, 0.223] | 1.022 |
| **Birth order (Second born)** |  |  | **-0.151*** | **[-0.294, -0.008]** |  | -0.142 | [-0.291, 0.008] |  |
| Birth order (Third or fourth born) |  |  | -0.369 | [-0.844, 0.105] |  | -0.189 | [-0.703, 0.325] |  |
| Multilingual household (Yes) |  |  | -0.034 | [-0.177, 0.109] | 1.004 | -0.093 | [-0.242, 0.056] | 1.021 |
| **Sex (Girl)** |  |  |  |  |  | **0.410***** | **[0.299, 0.520]** | **1.010** |
| Preterm birth (Yes) |  |  |  |  |  | 0.731 | [-0.154, 1.616] | 1.096 |
| Admission to NICU at birth (Yes) |  |  |  |  |  | -0.108 | [-0.301, 0.084] | 1.060 |
| Twin birth (Yes) |  |  |  |  |  | -0.213 | [-0.593, 0.167] | 1.043 |
| Family member with delayed talking (Yes) |  |  |  |  |  | -0.168 | [-0.336, 3.2x10^-4^] | 1.012 |
| **Living area (urban)** |  |  |  |  |  | **-0.223***** | **[-0.366, -0.099]** | **1.018** |
| **Living area (countryside)** |  |  |  |  |  | **-0.272***** | **[-0.412, -0.132]** |  |
| **Mother’s age at partus** |  |  |  |  |  | **-0.022**** | **[-0.035, -0.009]** | **1.052** |
| **Pregnancy length** |  |  |  |  |  | **0.009**** | **[0.003, 0.015]** | **1.144** |
| Mother’s education (university) |  |  |  |  |  | 0.109 | [-0.054, 0.272] | 1.081 |
| Mother’s employment (full-time) |  |  |  |  |  | -0.009 | [-0.283, 0.266] | 1.029 |
| Mother’s employment (part-time or on leave) |  |  |  |  |  | -0.018 | [-0.302, 0.266] |  |
| Mother’s history of smoking (Yes) |  |  |  |  |  | 0.120 | [-0.007, 0.247] | 1.039 |
| * p < .05, ** p < .01, *** p < .001. β is unstandardized.  Birth order: reference, firstborn and single child is the reference. Living area: big city is the reference.  Abbreviations: EPDS, The Edinburgh postnatal depression scale; NICU, neonatal intensive care unit; CI, confidence interval; VIF, variance inflation factor. | | | | | | | | |

**Table S5.** Summary of multivariable linear regression of depressive symptoms across pregnancy and postpartum (average score) and toddler expressive language development

| Variable | Model 1 | | Model 2 | | | Model 3 | | |
| --- | --- | --- | --- | --- | --- | --- | --- | --- |
|  | F(1, 1758) = 0.031, p = 0.860, R^2^ = 0 | | **F(6, 1558) = 2.339, p = 0.030, R^2^ = 0.009** | | | **F(19, 1464) = 6.790, p < .001, R^2^ = 0.081** | | |
|  | β | 95% CI | β | 95% CI | VIF | β | 95% CI | VIF |
| Mean of EPDS score (pregnancy and postpartum) | 0.001 | [-0.014, 0.017] | 0.007 | [-0.009, 0.024] | 1.107 | 0.006 | [-0.011, 0.024] | 1.142 |
| Maternal bonding difficulties (6 months postpartum) |  |  | -0.005 | [-0.014, 0.004] | 1.102 | -0.005 | [-0.014, 0.004] | 1.115 |
| Birth order (First born with other siblings) |  |  | 0.134 | [-0.013, 0.282] | 1.003 | 0.069 | [-0.080, 0.219] | 1.022 |
| **Birth order (Second born)** |  |  | **-0.175*** | **[-0.324, -0.025]** |  | -0.141 | [-0.291, 0.010] |  |
| Birth order (Third or fourth born) |  |  | -0.282 | [-0.791, 0.227] |  | -0.191 | [-0.734, 0.463] |  |
| Multilingual household (Yes) |  |  | -0.044 | [-0.193, 0.104] | 1.004 | -0.086 | [-0.236, 0.064] | 1.022 |
| **Sex (Girl)** |  |  |  |  |  | **0.405***** | **[0.294, 0.517]** | **1.010** |
| Preterm birth (Yes) |  |  |  |  |  | 0.698 | [-0.239, 1.635] | 1.081 |
| Admission to NICU at birth (Yes) |  |  |  |  |  | -0.079 | [-0.274, 0.116] | 1.057 |
| Twin birth (Yes) |  |  |  |  |  | -0.203 | [-0.582, 0.176] | 1.046 |
| Family member with delayed talking (Yes) |  |  |  |  |  | -0.160 | [-0.329, 0.008] | 1.012 |
| **Living area (urban)** |  |  |  |  |  | **-0.237***** | **[-0.372, -0.103]** | **1.018** |
| **Living area (countryside)** |  |  |  |  |  | **-0.279***** | **[-0.420, -0.138]** |  |
| **Mother’s age at partus** |  |  |  |  |  | **-0.023**** | **[-0.037, -0.010]** | **1.052** |
| **Pregnancy length** |  |  |  |  |  | **0.010**** | **[0.004, 0.016]** | **1.129** |
| Mother’s education (university) |  |  |  |  |  | 0.118 | [-0.046, 0.281] | 1.083 |
| Mother’s employment (full-time) |  |  |  |  |  | -0.054 | [-0.333, 0.225] | 1.032 |
| Mother’s employment (part-time or on leave) |  |  |  |  |  | -0.054 | [-0.342, 0.234] |  |
| Mother’s history of smoking (Yes) |  |  |  |  |  | 0.128 | [-2.75x10^-4^, 0.256] | 1.043 |
| * p < .05, ** p < .01, *** p < .001. β is unstandardized.  Birth order: reference, firstborn and single child is the reference. Living area: big city is the reference.  Abbreviations: EPDS, The Edinburgh postnatal depression scale; NICU, neonatal intensive care unit; CI, confidence interval; VIF, variance inflation factor. | | | | | | | | |

**Table S6.** Summary of multivariable linear regression of perinatal depressive symptoms (4 time points) and toddler expressive language development (Girls, Model 3 only)

| **Coefficients** | | | | | | | | | | | | | | | | | | | | | |  |  |
| --- | --- | --- | --- | --- | --- | --- | --- | --- | --- | --- | --- | --- | --- | --- | --- | --- | --- | --- | --- | --- | --- | --- | --- |
|  | | | | | | | | | | | | | | **95% CI** | | | **Collinearity Statistics** | | | | |  |  |
| **Model** | |  | | **Unstandardized** | | **Standard Error** | | **Standardizedᵃ** | | **t** | | **p** | | **Lower** | **Upper** | | **Tolerance** | | **VIF** | | |  |  |
| H₀ |  | (Intercept) |  | 3.437 |  | 0.040 |  |  |  | 86.843 |  | < .001 |  | 3.359 |  | 3.515 |  |  | |  |  | |  |
| H₁ |  | (Intercept) |  | 0.989 |  | 1.217 |  |  |  | 0.812 |  | 0.417 |  | -1.401 |  | 3.378 |  |  | |  |  | |  |
|  |  | **EPDS 17th week of pregnancy** |  | **0.025** |  | **0.012** |  | **0.101** |  | **2.080** |  | **0.038** |  | **0.001** |  | **0.049** |  | **0.724** | |  | **1.381** | |  |
|  |  | EPDS 32nd week of pregnancy |  | 0.003 |  | 0.013 |  | 0.014 |  | 0.265 |  | 0.791 |  | -0.022 |  | 0.029 |  | 0.683 | |  | 1.465 | |  |
|  |  | EPDS 6 weeks postpartum |  | -0.017 |  | 0.011 |  | -0.071 |  | -1.488 |  | 0.137 |  | -0.040 |  | 0.005 |  | 0.733 | |  | 1.365 | |  |
|  |  | EPDS 6 months postpartum |  | -0.002 |  | 0.011 |  | -0.008 |  | -0.169 |  | 0.865 |  | -0.024 |  | 0.020 |  | 0.748 | |  | 1.336 | |  |
|  |  | Maternal bonding difficulties (6 months postpartum) |  | -0.002 |  | 0.007 |  | -0.012 |  | -0.297 |  | 0.767 |  | -0.015 |  | 0.011 |  | 0.899 | |  | 1.113 | |  |
|  |  | Birth order (First born with other siblings) |  | 0.029 |  | 0.109 |  |  |  | 0.268 |  | 0.789 |  | -0.184 |  | 0.242 |  | 0.970 | |  | 1.031 | |  |
|  |  | Birth order (Second born) |  | -0.153 |  | 0.109 |  |  |  | -1.412 |  | 0.158 |  | -0.367 |  | 0.060 |  |  | |  |  | |  |
|  |  | Birth order (Third or fourth born) |  | -0.099 |  | 0.355 |  |  |  | -0.279 |  | 0.781 |  | -0.796 |  | 0.598 |  |  | |  |  | |  |
|  |  | Multilingual household (Yes) |  | -0.048 |  | 0.107 |  |  |  | -0.448 |  | 0.654 |  | -0.259 |  | 0.163 |  | 0.966 | |  | 1.035 | |  |
|  |  | Preterm birth (Yes) |  | 0.991 |  | 0.691 |  |  |  | 1.434 |  | 0.152 |  | -0.366 |  | 2.348 |  | 0.902 | |  | 1.109 | |  |
|  |  | Admission to NICU (Yes) |  | -0.143 |  | 0.150 |  |  |  | -0.954 |  | 0.340 |  | -0.437 |  | 0.151 |  | 0.920 | |  | 1.086 | |  |
|  |  | Twin birth (Yes) |  | -0.273 |  | 0.257 |  |  |  | -1.065 |  | 0.287 |  | -0.777 |  | 0.231 |  | 0.951 | |  | 1.051 | |  |
|  |  | **Family history in language delay (Yes)** |  | -0.281 |  | 0.121 |  |  |  | -2.316 |  | 0.021 |  | -0.519 |  | -0.043 |  | 0.978 | |  | 1.022 | |  |
|  |  | **Living area (small urban area)** |  | -0.217 |  | 0.094 |  |  |  | -2.304 |  | 0.022 |  | -0.402 |  | -0.032 |  | 0.972 | |  | 1.028 | |  |
|  |  | **Living area (countryside)** |  | -0.279 |  | 0.102 |  |  |  | -2.728 |  | 0.007 |  | -0.480 |  | -0.078 |  |  | |  |  | |  |
|  |  | **Mother’s age at partus** |  | -0.022 |  | 0.009 |  | -0.088 |  | -2.393 |  | 0.017 |  | -0.040 |  | -0.004 |  | 0.954 | |  | 1.048 | |  |
|  |  | **Pregnancy length** |  | 0.013 |  | 0.004 |  | 0.125 |  | 3.061 |  | 0.002 |  | 0.005 |  | 0.021 |  | 0.860 | |  | 1.162 | |  |
|  |  | Mother’s education (university) |  | 0.021 |  | 0.117 |  |  |  | 0.176 |  | 0.861 |  | -0.209 |  | 0.250 |  | 0.915 | |  | 1.093 | |  |
|  |  | Mother’s employment (full-time) |  | -0.271 |  | 0.190 |  |  |  | -1.426 |  | 0.154 |  | -0.644 |  | 0.102 |  | 0.965 | |  | 1.037 | |  |
|  |  | Mother’s employment (part-time or on leave) |  | -0.213 |  | 0.196 |  |  |  | -1.086 |  | 0.278 |  | -0.598 |  | 0.172 |  |  | |  |  | |  |
|  |  | Mother’s history of smoking (Yes) |  | 0.113 |  | 0.089 |  |  |  | 1.279 |  | 0.201 |  | -0.061 |  | 0.287 |  | 0.952 | |  | 1.051 | |  |
|  | | | | | | | | | | | | | | | | | | | | | |  |  |
| ᵃ Standardized coefficients can only be computed for continuous predictors.  Model 3: F(21, 754) = 2.709, p < 0.001, R^2^ = 0.070 | | | | | | | | | | | | | | | | | | | | | |  |  |

Abbreviations: EPDS, The Edinburgh postnatal depression scale; NICU, neonatal intensive care unit; CI, confidence interval; VIF, variance inflation factor.

**Table S7.** Summary of multivariable linear regression of perinatal depressive symptoms (4 time points) and toddler expressive language development (Boys, Model 3 only)

| **Coefficients** | | | | | | | | | | | | | | | | | | | | | |
| --- | --- | --- | --- | --- | --- | --- | --- | --- | --- | --- | --- | --- | --- | --- | --- | --- | --- | --- | --- | --- | --- |
|  | | | | | | | | | | | | | | **95% CI** | | | | **Collinearity Statistics** | | | |
| **Model** | |  | | **Unstandardized** | | **Standard Error** | | **Standardizedᵃ** | | **t** | | **p** | | **Lower** | | **Upper** | | **Tolerance** | | **VIF** | |
| H₀ |  | (Intercept) |  | 3.027 |  | 0.039 |  |  |  | 77.328 |  | < .001 |  | 2.950 |  | 3.104 |  |  |  |  |  |
| H₁ |  | (Intercept) |  | 1.418 |  | 1.229 |  |  |  | 1.154 |  | 0.249 |  | -0.994 |  | 3.830 |  |  |  |  |  |
|  |  | EPDS 17th week of pregnancy |  | 0.005 |  | 0.013 |  | 0.018 |  | 0.387 |  | 0.699 |  | -0.020 |  | 0.030 |  | 0.724 |  | 1.381 |  |
|  |  | EPDS 32nd week of pregnancy |  | -0.010 |  | 0.013 |  | -0.040 |  | -0.811 |  | 0.418 |  | -0.035 |  | 0.015 |  | 0.703 |  | 1.423 |  |
|  |  | EPDS 6 weeks postpartum |  | 0.016 |  | 0.012 |  | 0.065 |  | 1.323 |  | 0.186 |  | -0.008 |  | 0.039 |  | 0.700 |  | 1.430 |  |
|  |  | EPDS 6 months postpartum |  | -0.003 |  | 0.012 |  | -0.012 |  | -0.256 |  | 0.798 |  | -0.026 |  | 0.020 |  | 0.722 |  | 1.386 |  |
|  |  | Maternal bonding difficulties (6 months postpartum) |  | -0.003 |  | 0.006 |  | -0.017 |  | -0.426 |  | 0.670 |  | -0.015 |  | 0.010 |  | 0.863 |  | 1.159 |  |
|  |  | Birth order (First born with other siblings) |  | 0.024 |  | 0.102 |  |  |  | 0.236 |  | 0.814 |  | -0.177 |  | 0.225 |  | 0.969 |  | 1.032 |  |
|  |  | Birth order (Second born) |  | -0.204 |  | 0.105 |  |  |  | -1.952 |  | 0.051 |  | -0.410 |  | 0.001 |  |  |  |  |  |
|  |  | Birth order (Third or fourth born) |  | -0.341 |  | 0.378 |  |  |  | -0.902 |  | 0.367 |  | -1.082 |  | 0.401 |  |  |  |  |  |
|  |  | Multilingual household (Yes) |  | -0.115 |  | 0.106 |  |  |  | -1.082 |  | 0.280 |  | -0.323 |  | 0.094 |  | 0.978 |  | 1.023 |  |
|  |  | Preterm birth (Yes) |  | 0.356 |  | 0.524 |  |  |  | 0.679 |  | 0.497 |  | -0.672 |  | 1.384 |  | 0.859 |  | 1.164 |  |
|  |  | Admission to NICU (Yes) |  | -0.066 |  | 0.127 |  |  |  | -0.517 |  | 0.605 |  | -0.316 |  | 0.184 |  | 0.934 |  | 1.071 |  |
|  |  | Twin birth (Yes) |  | -0.222 |  | 0.253 |  |  |  | -0.877 |  | 0.381 |  | -0.718 |  | 0.274 |  | 0.961 |  | 1.040 |  |
|  |  | Family history in language delay (Yes) |  | -0.082 |  | 0.118 |  |  |  | -0.693 |  | 0.489 |  | -0.314 |  | 0.150 |  | 0.975 |  | 1.026 |  |
|  |  | **Living area (small urban area)** |  | **-0.239** |  | **0.096** |  |  |  | **-2.482** |  | **0.013** |  | **-0.429** |  | **-0.050** |  | **0.974** |  | **1.027** |  |
|  |  | **Living area (countryside)** |  | **-0.285** |  | **0.098** |  |  |  | **-2.917** |  | **0.004** |  | **-0.477** |  | **-0.093** |  |  |  |  |  |
|  |  | **Mother’s age at partus** |  | **-0.023** |  | **0.009** |  | **-0.089** |  | **-2.430** |  | **0.015** |  | **-0.042** |  | **-0.004** |  | **0.944** |  | **1.059** |  |
|  |  | Pregnancy length |  | 0.007 |  | 0.004 |  | 0.073 |  | 1.760 |  | 0.079 |  | -8.317×10^-4^ |  | 0.015 |  | 0.836 |  | 1.196 |  |
|  |  | Mother’s education (university) |  | 0.225 |  | 0.117 |  |  |  | 1.928 |  | 0.054 |  | -0.004 |  | 0.454 |  | 0.910 |  | 1.099 |  |
|  |  | Mother’s employment (full-time) |  | 0.319 |  | 0.213 |  |  |  | 1.500 |  | 0.134 |  | -0.098 |  | 0.737 |  | 0.961 |  | 1.040 |  |
|  |  | Mother’s employment (part-time or on leave) |  | 0.298 |  | 0.218 |  |  |  | 1.363 |  | 0.173 |  | -0.131 |  | 0.726 |  |  |  |  |  |
|  |  | Mother’s history of smoking (Yes) |  | 0.090 |  | 0.093 |  |  |  | 0.967 |  | 0.334 |  | -0.093 |  | 0.273 |  | 0.956 |  | 1.046 |  |
|  | | | | | | | | | | | | | | | | | | | | | |
| \| ᵃ Standardized coefficients can only be computed for continuous predictors.  Model 3: F(21, 791) = 2.265, p = 0.001, R^2^ = 0.057 \| \| --- \|   Abbreviations: EPDS, The Edinburgh postnatal depression scale; NICU, neonatal intensive care unit; CI, confidence interval; VIF, variance inflation factor. | | | | | | | | | | | | | | | | | | | | | |
